# Computer simulations of Template-Directed RNA Synthesis driven by temperature cycling in diverse sequence mixtures

**DOI:** 10.1371/journal.pcbi.1010458

**Published:** 2022-08-24

**Authors:** Pouyan Chamanian, Paul G. Higgs

**Affiliations:** 1 Origins Institute and Dept of Biology, McMaster University, Hamilton, Ontario, Canada; 2 Origins Institute and Dept of Physics and Astronomy, McMaster University, Hamilton, Ontario, Canada; University of Missouri, UNITED STATES

## Abstract

We present simulations of non-enzymatic template-directed RNA synthesis that incorporate primer extension, ligation, melting, and reannealing. Strand growth occurs over multiple heating/cooling cycles, producing strands of several hundred nucleotides in length, starting with random oligomers of 4 to 10 nucleotides. A strand typically grows by only 1 or 2 nucleotides in each cycle. Therefore, a strand is copied from many different templates, not from one specific complementary strand. A diverse sequence mixture is produced, and there is no exact copying of sequences, even if single base additions are fully accurate (no mutational errors). It has been proposed that RNA systems may contain a virtual circular genome, in which sequences partially overlap in a way that is mutually catalytic. We show that virtual circles do not emerge naturally in our simulations, and that a system initiated with a virtual circle can only maintain itself if there are no mutational errors and there is no input of new sequences formed by random polymerization. Furthermore, if a virtual sequence and its complement contain repeated short words, new sequences can be produced that were not on the original virtual circle. Therefore the virtual circle sequence cannot maintain itself. Functional sequences with secondary structures contain complementary words on opposite sides of stem regions. Both these words are repeated in the complementary sequence; hence, functional sequences cannot be encoded on a virtual circle. Additionally, we consider sequence replication in populations of protocells. We suppose that functional ribozymes benefit the cell which contains them. Nevertheless, scrambling of sequences occurs, and the functional sequence is not maintained, even when under positive selection.

## Author summary

The earliest form of RNA replication may have been non-enzymatic, without requiring polymerase ribozymes. Non-enzymatic replication forms double strands that are unlikely to separate unless melting is driven by temperature cycling. However, re-annealing of existing strands occurs rapidly on cooling, and this prevents subsequent cycles of copying if there are multiple copies of similar sequences. In contrast, if there is a diverse mixture of sequences, partially matching sequences can reanneal in configurations that allow continued strand growth. We show that this allows continued synthesis of populations of random sequences that are quite long. We test the idea that a virtual circular genome could exist in such a mixture. We show that a virtual genome does not arise spontaneously and that it cannot be maintained except in unrealistic ideal cases. We conclude that functional sequence information cannot be encoded on the fragments of a virtual circle.

## Introduction

It is widely believed that there was an RNA World period in the early stages of life on Earth in which RNA sequences played the roles of both genes and catalysts [1–5]. RNA replication in the RNA World may have been catalyzed by polymerase ribozymes, and there has been considerable progress in developing functional polymerase ribozymes in the laboratory [6–10]. However, it also seems likely that some form of non-enzymatic template-directed synthesis could have preceded the origin of polymerase ribozymes. The rates of nonenzymatic primer-extension have been measured in the laboratory [11–12]. It is found to be quite slow and error prone. Furthermore, a stalling effect is observed [13], such that the rate of addition after a previous error is very much slower than after a correctly matching pair, and the probability of another error after a previous error is higher. There are a number of practical problems associated with non-enzymatic replication [14], but there has been continued progress by making use of new forms of activated nucleotides and using downstream oligomers to stabilize the association of the incoming base with the growing primer and template [15–17].

One of the most important problems with non-enzymatic replication is product inhibition. Synthesis of complementary strands leads to the formation of double strands which are stable and difficult to separate, and which inhibit further replication. This has led to the suggestion that temperature cycling could enable repeated rounds of sequence replication: double strands would separate at high temperature, and each of these strands would be a template during the next low-temperature phase. However, reannealing of existing strands is likely to be fast compared to synthesis of new complementary strands. We have modelled the temperature cycling process [18] and shown that reannealing prevents replication once a limiting strand concentration is reached when reannealing becomes faster than synthesis of new strands. The limiting concentration is very low if the polymerization rate is slow, as we expect from measurements of non-enzymatic replication.

We therefore argued [18] for an alternative mechanism involving strand-displacement, in which an old complementary strand is displaced in a stepwise manner as a new complementary strand is synthesized. This can be done at constant temperature without the need for cycling. Many viruses replicate by strand-displacement driven by a protein polymerase, and there is also some experimental progress on non-enzymatic strand displacement [19]. One mechanism that seems likely as a means of starting replication in the RNA World is rolling circle replication [18], which is strand displacement on a circular template. This avoids a problem that occurs on a linear template, where partially synthesized complementary strands are displaced from the template by a full complementary strand that is already present, thus preventing new complete strands from being formed. The smallest known RNA replicators in current biology are circular viroids [20] that replicate via a protein-catalyzed rolling circle mechanism. Rolling circle replication using a ribozyme catalyst has also recently been demonstrated in the laboratory [21].

Despite our arguments that reannealing inhibits replication via the temperature cycling mechanism [18], there are reasons to believe that temperature cycling can still allow non-enzymatic synthesis of long RNA strands in some circumstances. We previously considered only a simple case in which there were multiple copies of a single type of complementary plus and minus strand. In this case, reannealing creates a double strand which is inactive for further primer extension and replication. However, in a diverse mixture of strands with different sequences, there are many ways in which partially complementary strands can reanneal. If the 3’ end of one strand is paired in the middle of another strand, then continued primer extension can occur in a productive way, as has been pointed out by Zhou et al. [22]. The aim of the present paper is to develop a realistic computer simulation of primer extension incorporating temperature cycling and reannealing in order to test whether synthesis of long strands is possible.

Zhou et al. [22] have termed this mechanism the ‘virtual circular genome’. They envisage that many different short RNA sequences could exist in a population that are partially overlapping in such a way that they could be assembled into a circular strand. The complete circle need not exist in any one single strand. They argue that in this case, the collection of fragments would act as mutual templates for each other and that the whole system could replicate. It was further argued in [22] that the virtual genome might encode fragments of ribozymes that could combine via secondary structure formation to give complete functional ribozymes without the need for the whole ribozyme sequence to exist in a single sequence. This idea has been partly validated experimentally by Wachowius *et al*. [23], who showed that a functional polymerase ribozyme could be assembled from fragments by using complementary fragments as splints. Although this demonstrates that ligation is possible, it does not demonstrate continued replication of the fragments. We will return to this point in the results below, where we use computer simulations to test for assembly of a complete ribozyme from fragments and also for replication of the fragments.

The possibility of a virtual circular genome is intriguing and we wished to test it in this paper. There are two separate claims of [22] that must be distinguished. Firstly, there is the claim that in a mixture of fragments of different sequences, the sequences could act as mutual templates in such a way that continued synthesis of a population of strands of quite high lengths is maintained. Secondly, there is the claim that in such a mixture, replication of a virtual genome would occur, and that such a genome could encode functional ribozymes. Our results below support the first of these claims but not the second. We will show that in a population of diverse fragments there are many ways in which pairing can occur between strands in a way that is productive for further primer extension. This leads to scrambling of sequences and loss of sequence information.

Several previous simulation studies of template directed replication have been made, but these have used simplified models of the process. We previously studied a model for templated ligation [24] in which an oligomer can catalyze the ligation of two other shorter oligomers that are complementary to it. This ligation was treated as a single reaction step in the model, rather than treating binding and separation as separate steps. This model makes the interesting prediction that templating selects for uniform polymers that are good templates, resulting in the emergence of homochirality, regioselectivity in the polymer backbone, and a small subset of RNA nucleotides from a diverse mixture of similar possible monomers. Another templating model at this level was studied in [25]. Protein catalyzed template-directed ligation of random 12-mers was studied experimentally and simulated in [26]. Another detailed model of ligation was studied in [27] which predicts interesting features of the distribution of strand lengths, but which neglects the sequences of the strands. None enzymatic replication with explicit RNA sequences was also simulated in [28], although it was assumed that replication of each strand began from a primer at the beginning of each strand and proceeded in one cycle to the end. There was no reannealing of existing strands. None of these previous models includes the realistic separate steps of annealing, primer extension and ligation and the requirement for matching of complementary sequences in the way that we do in this paper.

## Methods

We carry out stochastic simulations of RNA synthesis in a solution containing monomers and RNA strands. At any given point, the program stores the sequences of all strands present, the positions of all helices connecting strands, and the number of available single monomers of each type of nucleotide. Allowed reaction steps include (i) nucleation of new oligomers from monomers on single-stranded templates; (ii) annealing of complementary single stranded regions to form helices; (iii) monomer addition (primer extension) on the 3’ ends of sequences that are paired to a template; (iv) ligation of two neighbouring strands that are bound to the same template; (v) melting of existing helices. Rates of all possible reaction steps are calculated, and the Gillespie algorithm [29] is used to select one possible reaction step with a probability proportional to its rate. All allowed processes have some possibility of happening, but those with the faster rates occur more often. This is a standard method for simulating stochastic reaction systems in chemistry. The time is augmented by a random amount δt with a mean equal to the inverse of the sum of the rates of all possible processes. The growth phase of the simulation proceeds for a fixed time duration (nominally 6 hours), after which there is a high temperature phase in which all strands are separated. Inflow and outflow of monomers and strands can then occur, after which a new growth phase is initiated. We follow the properties of the sequences over multiple complete cycles.

### Initial supply of monomers and oligomers

The system begins with an initial number of monomers (*N*_0_ = 5000) of each nucleotide A, C, G and U (*i*.*e* 20000 in total), and an initial number (*N*_*init*_ = 500) of oligomers of lengths in the range 4–10. These oligomers are assumed to be generated by random polymerization without a template and consist of random sequences of the four nucleotides. We suppose the oligomer length *n* has an exponential distribution, with a probability distribution *P*(*n*) = *Aλ*^*n*−4^, where *λ* = 1/2 and *A* is the constant necessary to normalize the distribution. We cut off this distribution at a maximum of 10. In this way, whenever sequences longer than 10 are formed in the simulation, it is clear that they have been created by templating reactions, not random polymerization.

We assume that length *l*_*0*_ = 4 is the shortest length of primer that is stable long enough for primer extension to occur. Dimers and trimers will also be present in the mixture, but we ignore them in this simulation because we assume that their rate of detaching from a template will be high, and that detaching is likely to occur before addition of new monomers to the primer. If dimers and trimers were included, there would be many on and off reaction steps that would make little difference but would slow the program down considerably. The mean length of the initial oligomers is

$$\bar{n}=\frac{\sum_{n=4}^{n=10}n\lambda^{n-4}}{\sum_{n=4}^{n=10}\lambda^{n-4}}=4.94.$$

### Nucleation of new oligomers from monomers

Strands in the mixture may be connected by double stranded regions (helices). New helices may be formed by nucleation of monomers on an existing strand, or by annealing two existing strands with complementary sequences. The minimum allowed helix length is *l*_*0*_ = 4. The probability of a new helix nucleating on a given strand is proportional to the number of unpaired windows of length *l*_*0*_ on this strand. At the beginning of each cycle when there are no other helices already present, a sequence of length *n* has a number of windows *w* = *n*−*l*_0_+1. This number is reduced when helices form because each helix blocks the formation of further overlapping helices. The program keeps track of the number of available windows on each strand. For each available window, the nucleation rate is

$$r_{nuc}=k_{nuc}\frac{N_{i1}N_{i2}N_{i3}N_{i4}}{N_{0}^{4}},$$

where *k*_*nuc*_ is the nucleation rate constant, and *N*_*i*1_, *N*_*i*2_, *N*_*i*3_ and *N*_*i*4_ are the number of available free monomers of the required type to form the new tetramer. The tetramer is assumed to be exactly complementary to the template strand. At the beginning of the simulation, the number of each monomer in the system is *N*_*0*_. Thus, according to equation 1, the nucleation rate is equal to *k*_*nuc*_ per window when the monomers are at their initial concentration, and it decreases in proportion to monomer concentration to the power 4 when the monomers are used up by the polymerization process. When a new tetramer is formed, the corresponding monomers are removed from the count of free monomers.

### Annealing existing strands

To form a new helix by annealing existing strands, there must be an available window of length *l*_*0*_ on two different strands. We define the attempted rate of annealing per pair of windows as *r*_*ann*_ = *k*_*ann*_/*N*_*o*_. For any given available window, let the total number of other windows on other strands that are potential partners for pairing be *w*_*tot*_. The total attempted rate of annealing to the first window is

$$r_{ann}^{tot}=k_{ann}\frac{w_{tot}}{N_{0}}.$$

There are initially *N*_*0*_ monomers of each type of nucleotide. If all these monomers were turned into tetramers, then *w*_*tot*_ would be *N*_*0*_. Thus by scaling the annealing rate by 1/*N*_*0*_ we keep the total attempted rate of annealing to one window proportional to the total concentration of sequence windows in the system. We choose random pairs of available windows at this attempt rate, but the annealing only occurs if the sequences are complementary. If a matching helix of the minimum length *l*_*0*_ is possible, the helix is formed in this position and then ‘zipped up’ in both directions as long as the bases in the two sequences form matching pairs and as long as further extension of the helix is not prevented by a previously existing helix.

### Monomer addition

**Fig 1:**
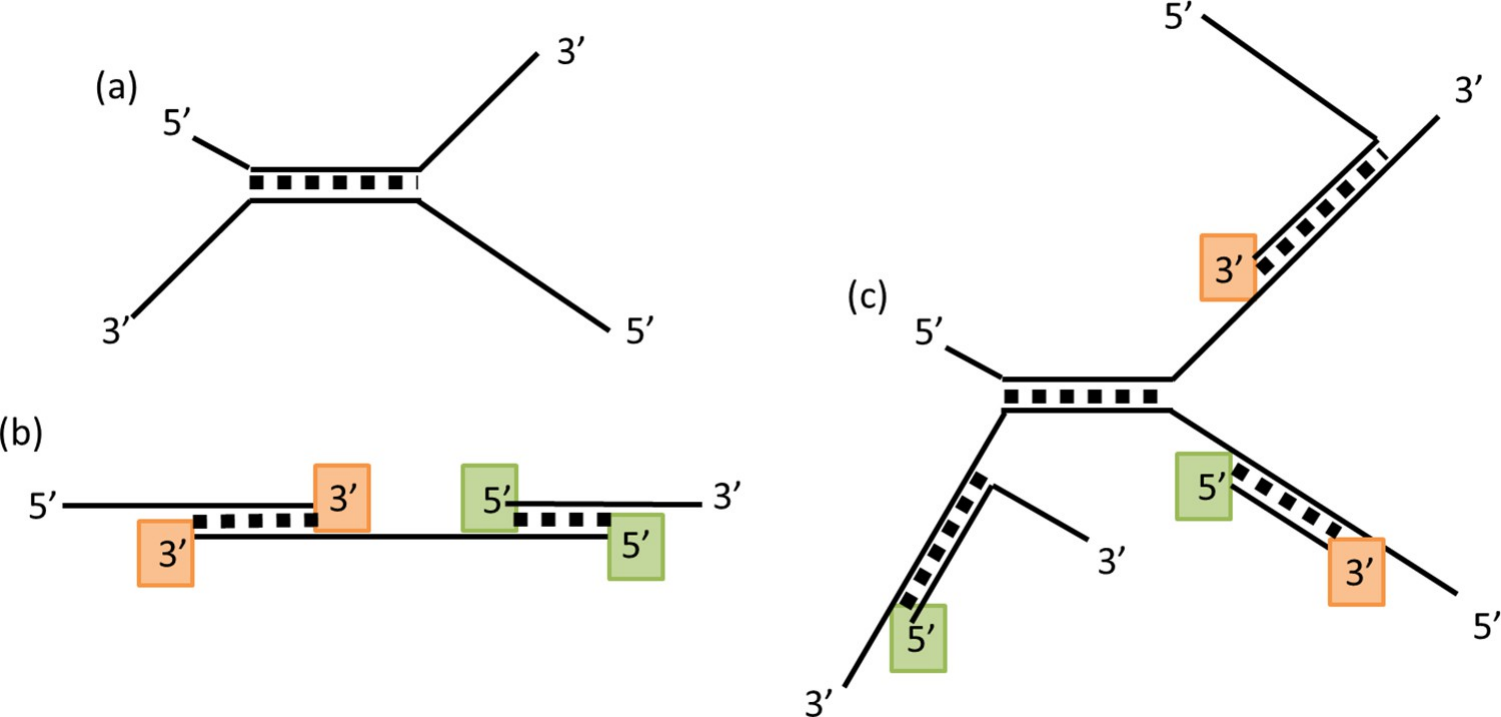
Examples of structures that form via annealing of strands. (a) If a helix forms in the middle of the strands, so that the ends of the strands are in single-stranded tails, then no growth is possible at these ends. (b) If the 3’ end of a strand is the last base in a helix (orange squares), this is a site for monomer addition. If the 5’ end of a strand is the last base in a helix (green squares), we assume that monomer addition cannot occur, but this is a potential site for ligation, if the 3’ end of another strand grows to be adjacent to this site. (c) Connection of multiple strands forms branching clusters with many tails and many potential points of sequence growth. We assume that formation of an additional helix is not possible between strands that are already in the same cluster. This prevents unrealistic loops and knots forming within a cluster.

If a helix forms in the middle of two sequences, the ends of the sequences are single-stranded tails which cannot grow (as in Fig 1A). When the 3’ end of a strand is at the end of a helix (as in Fig 1B and 1C, orange sites), monomer addition can occur (*i*.*e*. primer extension). Monomer addition is directional and only occurs at 3’ ends. When the 5’ end of a strand is at the end of a helix (as in Fig 1B and 1C, green sites), monomer addition cannot occur, but this 5’ end can be ligated to the 3’ end of another strand if the end of the other strand grows to be adjacent to this point.

Whenever there is a 3’ end that is paired and the next site on the template is unpaired, monomer addition (also known as primer extension) can occur at a rate

$$r_{add}=k_{add}\frac{N_{i}}{N_{0}},$$

where *N*_*i*_ is the number of monomers in solution of the type that are complementary to the template. We scale the rate by the initial number of monomers, *N*_*0*_. Thus, the addition rate is *k*_*add*_ when the monomer concentration is equal to the initial monomer concentration, and it decreases in proportion to the remaining concentration of free monomers.

Initially, we consider a basic rates model in which there is perfect pairing between template and the growing complementary strand, *i*.*e*. there is zero error rate. In this case, the only type of nucleotide that can be added is the one complementary to the template.

Secondly, we consider an equal-error-rates model in which a single error rate parameter ε determines the relative rate of addition of all non-Watson-Crick bases. The rate of addition of base *i* opposite template base *j* is

$$r_{add}(i|j)=k_{add}\frac{N_{i}}{N_{0}},\;\mathrm{if}\;i\;\mathrm{is}\;\mathrm{complementary}\;\mathrm{to}\;j,$$

$$r_{add}(i|j)=k_{add}\varepsilon \frac{N_{i}}{N_{0}},\;\mathrm{if}\;i\;\mathrm{is}\;\mathrm{not}\;\mathrm{complementary}\;\mathrm{to}\;j.$$

Thirdly, we consider a scaled-experimental-rates model in which rates for each combination are proportional to the rates measured in experiment. We make use of two sets of measured rates [11,12]. Table 1 shows the averages of these two sets of measurements. Both these experiments used a concentration of 10 mM of single nucleotides for A, C and G, but a concentration of 40 mM for U, because the rates of U addition are slow and increasing the concentration speeds up the experiment. We have adjusted for this in Table 1 by dividing all rates for addition of U by 4, which means that the rates in the table should correspond to an equal concentration of 10 mM for each base.

**Table 1:** Experimental rates of monomer addition *r*_*ex*_(*i*|*j*) in nucleotides per hour. Values are the averages of those measured by [11] and [12].

		Base added i			
		A	C	G	U
Template base j	A	0.043	0.079	0.145	0.204
	C	0.012	0.013	1.940	0.004
	G	0.037	14.515	0.205	0.174
	U	0.735	0.014	0.365	0.024

There is a large variation among the rates of addition of the four correct Watson-Crick pairs, and the average rate of these four is *r*_*WC*_ = 4.348 h^-1^. We scaled all the experimental rates by a factor $\frac{k_{add}}{r_{WC}}$ so that

$$r_{add}(i|j)=k_{add}\frac{r_{ex}(i|j)}{r_{WC}}\frac{N_{i}}{N_{0}}.$$

The average rate of addition of the Watson-Crick pairs is *k*_*add*_ after this scaling, which is directly comparable with the simpler models.

### Ligation

Whenever there is a 3’ end that is paired and the next site on the template is paired to the 5’ end of another strand, then ligation can occur at rate *k*_*lig*_. This rate is constant, independent of the concentration of monomers and strands.

We keep track of clusters of sequences that are connected by helices. At the beginning of each growth phase, each strand is defined to be in its own separate cluster. When two sequences are connected, they are placed in the same cluster. We impose the restriction that a new helix can be formed between two strands only if they are not already in the same cluster. This means that all the clusters that form have a branching tree structure (as in Fig 1C). In an early version of this program in which this restriction was not imposed, we found that multiple connections formed between sets of sequences that were already in the same cluster, creating a dense structure of entangled knots and loops. Such a structure would be impossible to achieve in three-dimensional space with real molecules because of excluded volume restrictions and the finite length and flexibility of strands. Our simulation does not account for excluded volume and three-dimensional coordinates of the strands. We impose the branching cluster rule as a simple way of preventing the formation of unrealistic loops and knotted structures.

### Helix melting

We suppose that polymerization and ligation occur during the growth phase of the cycle in which the temperature is moderate and the double strands are stable. The growth phase lasts for a time *T*_*grow*_ = 6 hours. At the end of the growth phase there is a high-temperature phase in which all helices are immediately melted independent of length. The melting temperature of long double helices may be quite high in practice, and may be difficult to drive purely by temperature increase. However, experiments on DNA ligation have shown [30] that melting of long double helices can occur at low salt content and low pH without requiring an unreasonably high temperature. He we simply assume that all helices melt at the end of each cycle, by some means.

Melting of individual helices can also occur at a finite rate during the growth phase of our simulations. When this occurs, the two strands forming a helix are separated, and the cluster containing the helix is divided into two separate clusters. The melting rate for a minimum-length helix of length *l*_*0*_ = 4 is *k*_*melt*_. The melting rate for a longer helix of length *l* is

$$r_{melt}(l)=k_{melt}\;\exp\left(-\frac{(l-l_{0})\Delta G}{kT}\right),$$

where ΔG is the average stacking free energy per additional base pair in the helix. For simplicity, we treat stacking free energy using a single average value, and we do not consider sequence-specific stacking parameters.

### Inflow and outflow

After all helices are melted, we deal with input and output to the system. We want to be able to demonstrate that continued synthesis of new strands is possible in the mixture. For practical computing reasons (memory and time) we cannot allow the number of sequences in the simulation to increase exponentially for ever. The simplest way to limit the number of sequences is by outflow. This could represent a physical system with a finite volume in which there is input and output of material by diffusion or by steady fluid flow. It is also similar to what would happen in a growing and dividing vesicle, since growth leads to dilution. The main point is to demonstrate that synthesis of new strands inside the reaction system is sufficient to replace strands that are lost due to dilution, flow, or cell division, and to look at the distribution of lengths that exists when the system reaches a steady state.

Most results in this paper deal with a single mixture of sequences with input and output driven by a flow rate *ϕ*. Each strand is lost from the system with a probability *ϕ* at the end of each cycle. A fraction *ϕ* of monomers of each type is lost from the system. New monomers and oligomers are then added at the initial concentration. Thus, *ϕN*_*0*_ monomers of each type are added, and a number *N*_*inflow =*_ *ϕN*_*init*_ of new oligomers are added with the same exponential length distribution as the initial sequences.

We also consider simulations where only monomers are supplied at each new cycle and there is no inflow of new oligomers. This would be the case if RNA synthesis was occurring inside lipid vesicles whose membrane is impermeable to oligomers.

At the end of the paper, we consider simulations of populations of cells, each containing separate mixtures of sequences. In this case, we assume that the monomer concentration remains constant because the membranes are permeable to monomers, but there is no input or output of new sequences. The growth and division of the cell gives dilution of strands, and there is no need for an additional loss process to maintain a finite strand concentration.

### Standard parameters

A list of parameters of the model is given in Table 2. It should be noted that all the rate constants have the same units of h^-1^. Some of these processes also depend on the monomer concentration, but we have included monomer concentrations in the way rates are calculated (as described above), so the rate constants do not have a concentration dependence. We have also expressed monomer concentrations as ratios of monomer numbers in the simulation relative to the initial number. If the monomer concentration in an experimental situation was 10 mM (as was the case in [11]), and the addition rate was 10 h^-1^ at this concentration, then the simulation represents a volume *V* such that *N*_0_/*V* = 10mM, and *k*_*add*_ is the addition rate at this initial concentration. In this way, we do not need to specify the volume, or the actual monomer concentration.

**Table 2:** Standard values of the parameters used in the simulations.

Parameter	Standard Value	Meaning
*N*_*0*_	5000	Initial number of monomers of each type (A, C, G and U)
*N*_*init*_	500	Initial number of random oligomers
*ϕ*	0.05	Fractional flow rate = fraction of strands lost at the end of each cycle
*N*_*inflow*_	25	Number of random oligomers flowing in per cycle
*l*_*0*_	4	Minimum possible length of primers and helices
*λ*	0.5	Parameter controlling exponential distribution of primer lengths
*k*_*nuc*_	0.1h^-1^	Nucleation rate constant per hour at the initial nucleotide concentration.
*k*_*ann*_	10 h^-1^	Annealing rate constant per hour per window pair
*k*_*add*_	10 h^-1^	Rate constant for monomer addition at the initial nucleotide concentration.
*k*_*lig*_	1 h^-1^	Ligation rate constant
*k*_*melt*_	1 h^-1^	Melting rate of a helix of length *l*_*0*_
*ΔG/kT*	2	Stacking free energy per base pair, relative to kT.
*T*_*grow*_	6 h	Length of growth phase

### Graph functions X, S and C

Zhou *et al*. [22] proposed that sequences in a mixture of replicating RNAs might form a virtual circular arrangement. In order to detect the possible presence of virtual circles in our simulations, we consider 5-letter words (5*-*mers) within the sequences formed in the simulation. We choose 5-mers because we have already set the minimal length of a stable helix to be *l*_*0*_ = 4. Hence the shortest length of a sequence that can act as a template is 5. The number of possible 5-mers is 4^5^ = 1024. Each word can be labelled by an integer from *i* from 0 to 1023. Let *f*_*i*_ be the frequency of word *i* in the mixture, counting all overlapping 5-mers in sequences of length 5 or longer.

We will say that a word *j* follows from word *i* if the first 4 letters of *j* are the last four letters of *i*. There are four possible following words from any given word, as there are four possibilities for the last nucleotide in *j*. For a given mixture of words, we define a transition matrix *T*_*ij*_ such that *T*_*ij*_ = 1 if word *j* follows word *i* and both *i* and *j* are present in the mixture, and *T*_*ij*_ = 0 otherwise. We say there is a path of length *n* steps from *i* to *j* if there is a series of *n* words that follow from each other that are all present in the mixture. We define a path matrix, such that $P_{ij}^{n}=1$ if such a path exists, and $P_{ij}^{n}=0$ if no such path exists. Clearly, $P_{ij}^{1}=T_{ij}$. The path matrices for larger numbers of steps can be calculated by iteration. $P_{ij}^{n+1}=1$, if $\sum_{k}P_{ik}^{n}T_{kj}>0$, otherwise $P_{ij}^{n+1}=0$.

For any given word *i*, the fraction of words in the mixture that are accessible by a path of length *n* is $\sum_{j}f_{j}P_{ij}^{n}$. We define the connectivity function *X*(*n*) as the probability that two randomly chosen words from the mixture are connected by a path of length *n*.


$$X(n)=\sum_{i}\sum_{j}f_{i}f_{j}P_{ij}^{n}$$

If $P_{ii}^{n}=1$, there is a circular path that returns to word *i* after *n* steps. We define the circularity function *C*(*n*) as the fraction of words in the mixture which are part of a circular path of *n* steps.

$$C(n)=\sum_{i}f_{i}P_{ii}^{n}$$

Furthermore, we define $P_{ii}^{*}=1$ if word *i* is part of a circular path of any length (*i*.*e*. there is at least one *n* for which $P_{ii}^{n}=1$). The fraction of words that are part of a circular path of any length is

$$p_{circ}=\sum_{i}f_{i}P_{ii}^{*}$$

We also determine to what extent a particular word specifies the word that follows it after *n* steps. We define the specificity of word *i* as *S*_*i*_(*n*) = 0 if there is no word accessible from *i* by a path of length *n*, and *S*_*i*_(*n*) = 1 if there is exactly 1 accessible word. If there is more than one accessible word,

$$S_{i}(n)=\frac{{\underset{j}{\max}f_{j}P_{ij}}^{n}}{\sum_{j}f_{j}P_{ij}^{n}}.$$

This is the relative frequency of the most common accessible word in comparison to the total frequency of all accessible words. We define the specificity function of the mixture, *S*(*n*), as the mean value of specificity of the words:

$$S(n)=\sum_{i}f_{i}S_{i}(n).$$

The functions *X*(*n*), *S*(*n*) and *C*(*n*) have distinctive shapes that indicate clearly whether a virtual circle is present, as we will show in the Results section.

### Word graphs

The relationship between the set of 5-mer words occurring in a simulation can also be illustrated by drawing word graphs, in the following way. Each node represents a 5-mer word that is present in the mixture. The size of the nodes is scaled to indicate word frequency. Words not present in the mixture are not drawn in the graph. A directed link (arrow) is drawn between two nodes whenever the second node is a word that follows from the first.

Nodes which are part of cyclic paths are coloured red. Intermediate nodes on non-cyclic paths are coloured grey. Source nodes (those which have no preceding words) are coloured green. Sink nodes (those which have no following words) are coloured blue. Nodes are labelled with the last nucleotide of the word that they represent. Previous nucleotides can be deduced by tracing the pathways backwards. In the case of source nodes, this is not possible. Therefore, source nodes are labelled with the full 5-mer sequence.

## Results

### Basic polymerization model

**Fig 2:**
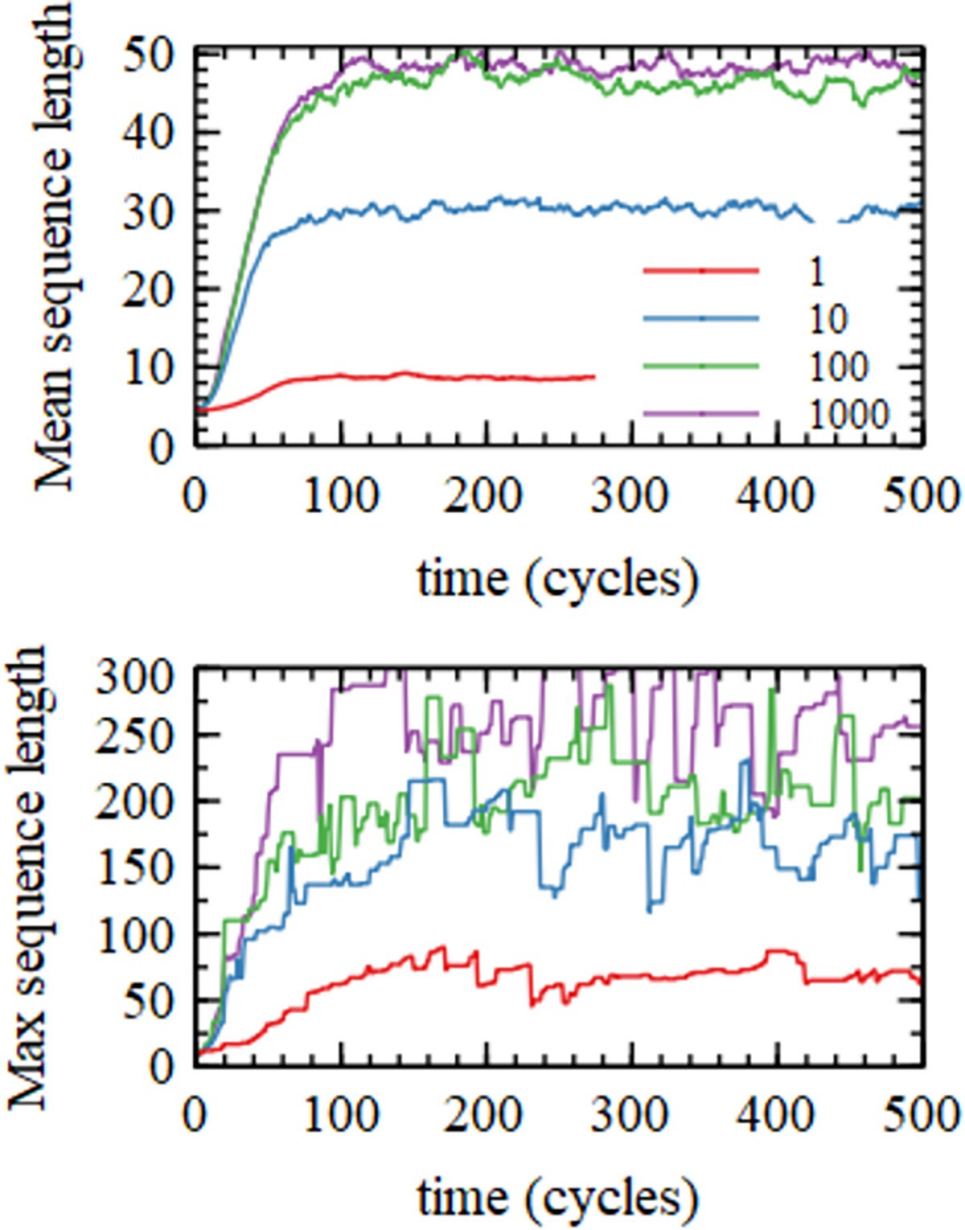
Mean length and Maximum length as a function of time in cycles for four simulations with varying values of the monomer addition rate *k*_*add*_.

**Fig 3:**
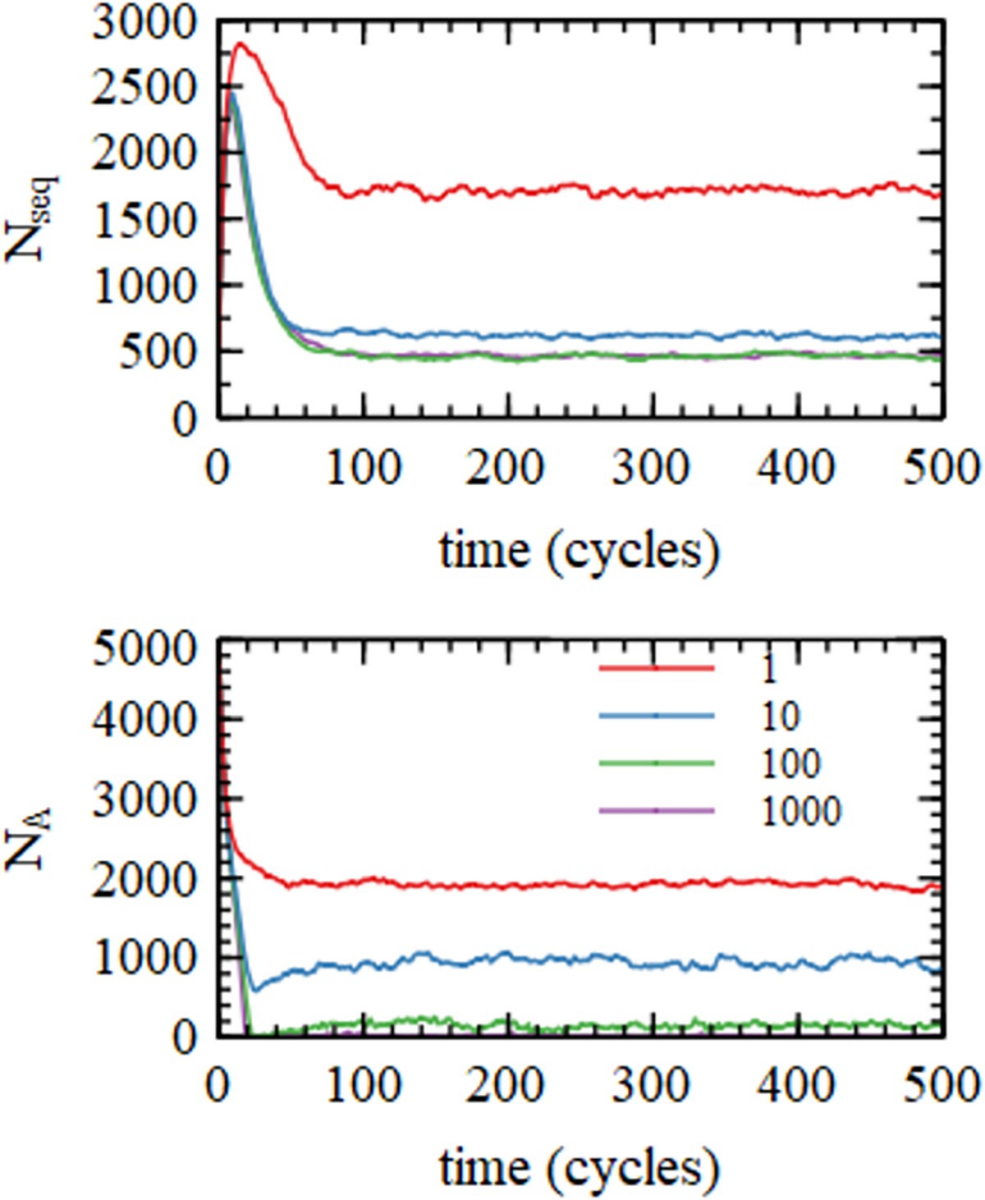
Number of sequences, *N*_*seq*_, and number of free A nucleotides, NA, as a function of time in cycles for four simulations with varying values of the monomer addition rate *k*_*add*_.

**Fig 4:**
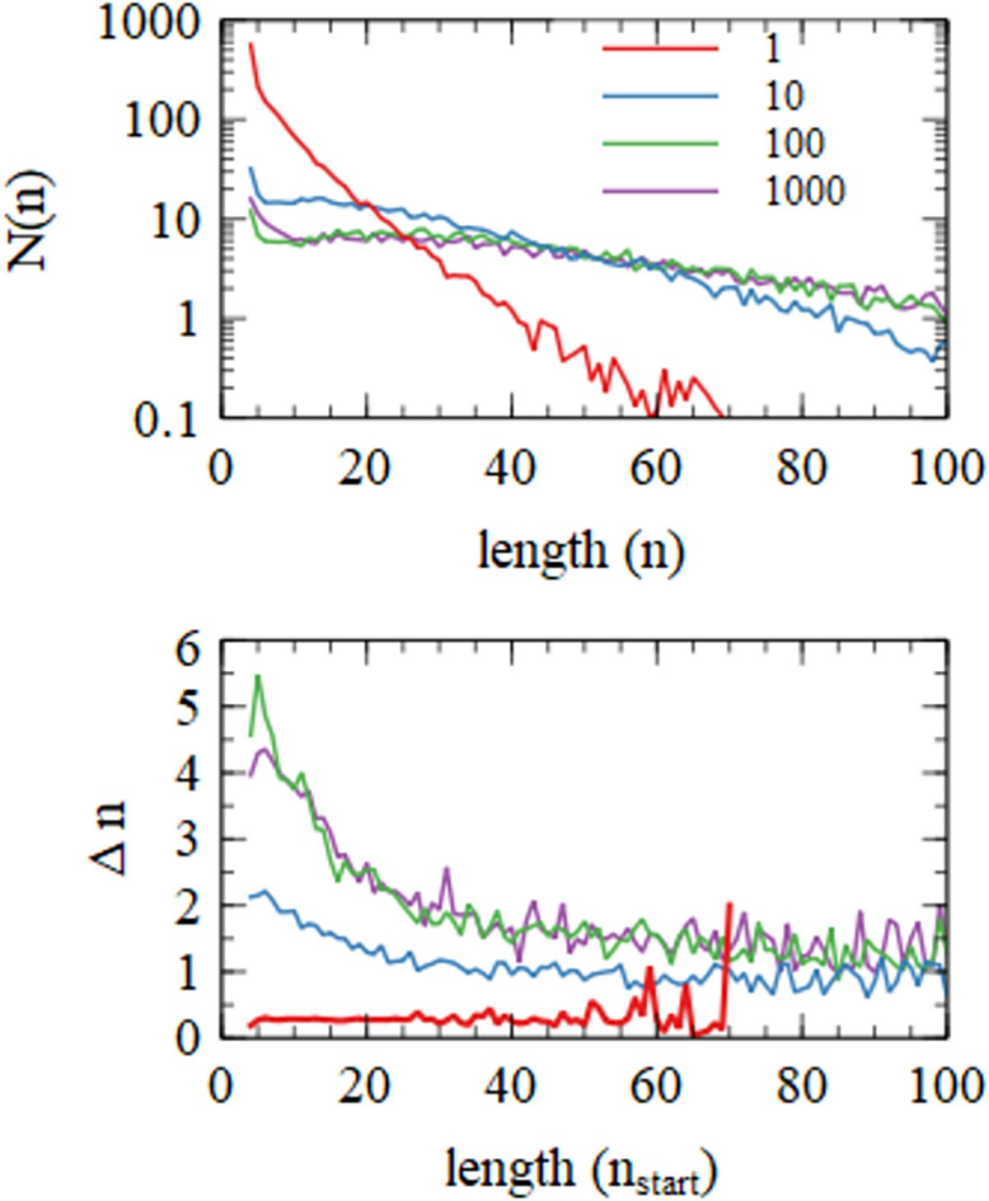
Distibution of sequence lengths, *N*(*n*), and mean increase in length, Δn, for four simulations with varying values of the monomer addition rate *k*_*add*_.

**Fig 5:**
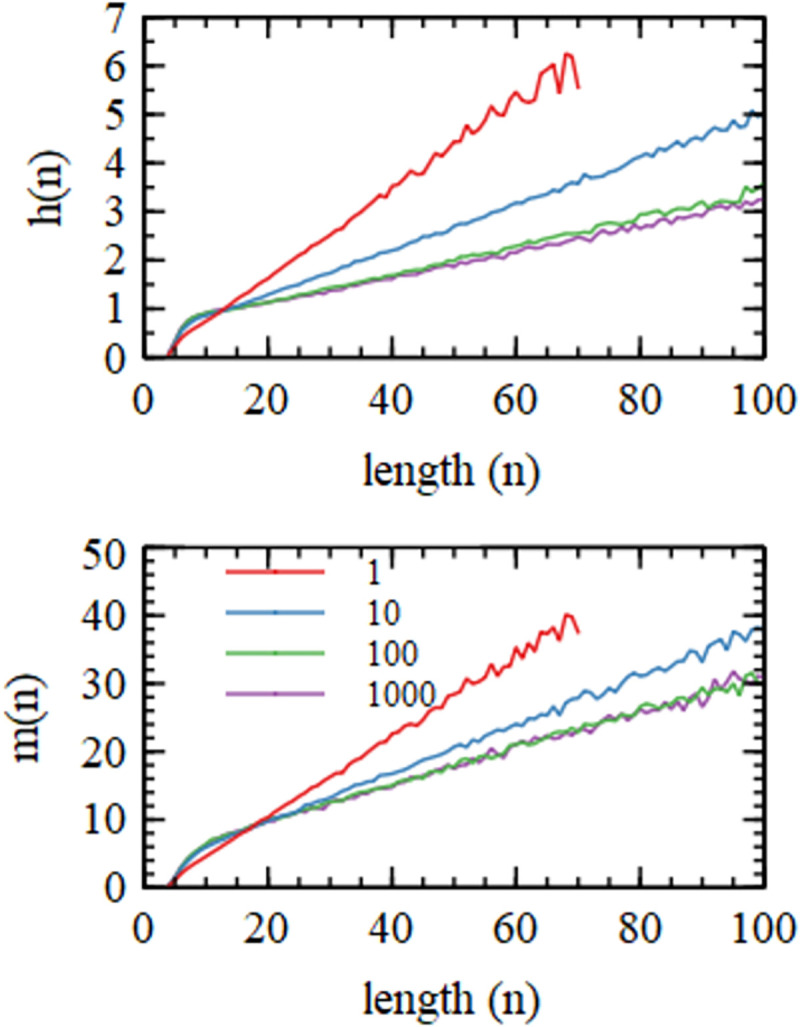
Mean number of helices, *h*(*n*), and mean number of paired bases, *m*(*n*), as a function of total sequence length *n* for four simulations with varying values of the monomer addition rate *k*_*add*_.

In this section we investigate the distribution of lengths of sequences formed when using the basic polymerization model. In this basic model there are no sequence errors. The only base that can be added to the growing strand is the correct Watson-Crick base that matches the template. Figs 2–5 show results from four separate simulations with four different values of the monomer addition rate *k*_*add*_. All other parameters take the standard values in Table 2. Quantities are shown as a function of time, with data plotted at the end of each growth phase, before melting of helices and loss of strands via outflow. In Fig 2, the mean length begins at the mean length of the inflowing oligomers ($\bar{n}$ = 4.94) and increases in time due to primer extension and ligation reactions. It reaches a stationary value at which the loss of old (long) strands is balanced by the inflow of new (short) oligomers. The mean length of sequences created by templating reactions is very much higher than *n*_*init*_. Fig 2 also shows the maximum length sequence in the population. This is substantially higher than the mean.

Fig 3 shows the number of sequences in the mixture, *N*_*seq*_. In each run, *N*_*seq*_ begins at the initial value *N*_*init*_ = 500. It rises rapidly in the first few cycles and then falls to a steady state value where the increase in strands due to inflow and nucleation is balanced by the outflow. The number of A monomers in the solution begins at *N*_*0*_ = 5000, and falls to a steady state value, where the reduction in monomers due to polymerization is balanced by the inflow rate *ϕN*_*0*_. The other three monomers, C, G and U, are close in number to A.

The number of strands in the mixture begins at *N*_*seq*_ = *N*_*init*_, and rises above this as new strands are nucleated from monomers. *N*_*seq*_ then falls again because the nucleation rate falls when the concentration of free monomers decreases. The steady state value of *N*_*seq*_ is highest for the lowest rate of monomer addition (*k*_*add*_ = 1 h^-1^) because the steady state monomer concentration remains quite high, and the nucleation rate remains high. For the highest rate of monomer addition (*k*_*add*_ = 1000), almost all monomers are polymerized, therefore the monomer concentrations fall almost to zero, and the nucleation rate becomes negligible. New sequences are then created only by inflow.

Fig 4 shows the steady state length distribution *N*(*n*). Lengths of each strand were taken at the end of the growth period of each cycle, and data were averaged over the last 250 cycles of the simulation runs, which lasted 500 cycles. The distribution of lengths arising from random polymerization is a rapidly decreasing exponential distribution (~*λ*^−*n*^). The measured distributions decrease with *n* very much more slowly than this (even for *k*_*add*_ = 1). For the three higher values of *k*_*add*_ there is a broad peak in the distribution, which clearly does not correspond to a single exponential. There are substantial numbers of long sequences in the range 50–100 nucleotides for all three of the larger values of *k*_*add*_ and small numbers of these sequences still exist even for the smallest *k*_*add*_.

For any given sequence, *n* is the length at the end of the growth phase, and *n*_*start*_ is the length of the same sequence at the start of the growth phase. The increase in length during one growth phase is Δ*n* = *n*−*n*_*start*_. Fig 4 shows the mean Δ*n* as a function of *n*_*start*_. It can be seen that Δ*n* is larger for higher *k*_*add*_, as would be expected, but Δ*n* is quite small, even for the largest addition rates. For *k*_*add*_ = 1000 h^-1^, Δ*n* is close to 5 for short sequences (tetramers and pentamers) and decreases to around 1 for longer sequences of length 100. For *k*_*add*_ = 1, Δ*n* is around 0.3, and is almost independent of *n* (large fluctuations at large *n* arise because of small numbers of sequences).

These results show that substantial numbers of long sequences are formed, even though the increase in length in any one cycle is quite small. Growth of long sequences thus requires continued presence in the reaction chamber over many cycles.

Fig 5 shows the mean number of separate helices *h(n)* in which a sequence is bound, as a function of the length of the sequence at the end of the growth period (averaged over the last 250 cycles). If *h* were 1 for every strand, the system would consist of paired strands only. If *h* were 2 for every strand, there would be a long chain of connected strands. If *h* were greater than 2 for every strand, there would be branching clusters. It is found that for short sequences *h(n)* is close to 1, and the number of helices increases linearly with *n*. A length *n =* 100 sequence has *h(n)* close to 3, for the fastest addition rate (*k*_*add*_ = 1000). This means that long sequences are involved in branching clusters that connect many strands (as in Fig 1C) rather than very long duplexes that link only two sequences. Fig 5 also shows the mean number of nucleotides, *m*(*n*), that are paired in helices at the end of the growth period, for a given total length *n*. It can be seen that *m*(*n*) is approximately linear with *n*, and that *m*(100) is around 30 for *k*_*add*_ = 1000. Hence, approximately 30% of nucleotides are paired.

Many simulations were carried out with varying values of the other rate constants. Table 2 shows a summary of measured values in these simulations. For each group of four runs, one rate is varied (as specified in column 1 of Table 3) and the other rates are fixed at their standard values given in Table 2. The runs with varying *k*_*add*_ are the same runs shown in Figs 2–5. In Table 3, the mean length, the number of sequences *N*_*seq*_ and the number of A nucleotides *N*_*A*_ are the values of these quantities at the end of growth phase of the last cycle (*t* = 500). The max length in the table is the maximum length of any sequence produced during the whole run. The functions Δ*n*(*n*) and *h*(*n*) were averaged over the final 250 cycles of the runs (as in Fig 4), and Table 3 summarizes this with the increase in length for short (*n* = 5) and longer sequences (*n* = 50) sequences. The number of helices and the number of paired bases are given for a sequence of length *n* = 50.

**Table 3:** Summary of quantities measured in simulation runs as a function of the rate variables. In each group of four runs, one rate constant is varied (as in the first column) and the other rates are set to their standard values in Table 1.

	mean length	max length	*N*_*seq*_	*N*_*A*_	Δ*n*(5)	Δ*n*(50)	*h*(50)	*m*(50)
*k*_*add*_ = 1 *k*_*add*_ = 10 *k*_*add*_ = 100 *k*_*add*_ = 1000	8.6	90	1703	1875	0.27	0.19	4.44	28.4
	31.0	232	603	870	2.15	0.87	2.70	20.7
	47.9	287	439	143	5.48	1.76	1.98	17.7
	46.1	300	464	5	4.30	1.41	1.86	17.5
*k*_*nuc*_ = 0 *k*_*nuc*_ = 0.01 *k*_*nuc*_ = 0.1 *k*_*nuc*_ = 1	37.1	267	456	1253	2.87	1.08	2.27	18.2
	35.1	258	507	1334	2.71	1.13	2.39	19.4
	31.0	232	603	870	2.15	0.87	2.70	20.7
	23.0	210	810	609	1.56	0.72	3.13	22.2
*k*_*ann*_ = 0 *k*_*ann*_ = 1 *k*_*ann*_ = 10 *k*_*ann*_ = 100	4.5	10	2997	2259	0.00	0.00	0.00	0.00
	18.4	191	994	1144	0.34	1.03	2.43	21.6
	31.0	232	603	870	2.15	0.87	2.70	20.7
	34.1	246	580	797	5.58	0.88	2.61	19.7
*k*_*melt*_ = 0 *k*_*melt*_ = 0.1 *k*_*melt*_ = 1 *k*_*melt*_ = 10	27.9	251	631	1138	2.22	0.80	3.21	21.2
	29.0	223	617	1070	2.16	0.81	3.02	20.5
	31.0	232	603	870	2.15	0.87	2.70	20.7
	23.4	247	712	1112	1.14	1.33	2.45	22.8

The trends with *k*_*add*_ have already been seen in Figs, 2–5. The mean and max lengths and Δ*n* all increase with *k*_*add*_ because there is a higher rate of primer extension. The concentration of available nucleotides decreases with *k*_*add*_, which causes a reduction in the nucleation rate and hence a decrease in the number of sequences. Δ*n* increases with *k*_*add*_, while *h*(*n*) and *m*(*n*) decrease.

We now consider the trends in Table 3 when the other rate parameters are varied. The rate of nucleation of new tetramer primers from monomers is proportional to *k*_*nuc*_ (equation 2). The second series in Table 2 shows that *N*_*seq*_ increases with *k*_*nuc*_ as expected. In the case *k*_*nuc*_ = 0, the only new strands created are random oligomers that enter on each cycle with the inflow. Increasing *k*_*nuc*_ leads to a decrease in the available number of free monomers, *N*_*A*_, and a decrease in the mean and max lengths (*i*.*e* a larger number of shorter sequences). Δ*n* decreases with *k*_*nuc*_, while *h*(*n*) and *m*(*n*) increase. It can be seen that the nucleation process is not essential to sustain replication, because a population of sequences can be sustained by inflow of random oligomers even when *k*_*nuc*_ = 0. However, the results are fairly sensitive to even a small rate of nucleation; therefore, this is an important parameter to include in the simulation.

The annealing rate *k*_*ann*_ is also an important parameter. When *k*_*ann*_ = 0, an existing sequence never binds to another existing sequence, and the only helices formed are between newly nucleated tetramers and existing oligomers. The new strand can never grow longer than the template strand. The mean length in this case is 4.5, which is slightly shorter than the mean of the random sequences in the inflow ($\bar{n}$ = 4.9), and the max length is 10, which is the max length in the random inflowing sequences. Thus, to grow long sequences, it is essential to have *k*_*ann*_ > 0. When *k*_*ann*_ increases, the probability of forming structures that allow productive primer extension and ligation increases. Therefore, the mean and max lengths increase with *k*_*ann*_ (third series of Table 3). *N*_*seq*_ decreases because when sequences anneal to existing strands, fewer single-stranded sites remain for nucleation of new sequences. The number of free monomers decreases with *k*_*ann*_ because annealing promotes primer extension, which uses up the monomers. When *k*_*ann*_ = 0, there is some probability of growth of a newly nucleated tetramer (we find Δ*n*(4) = 0.03), but longer sequences cannot grow without annealing; hence Δ*n*(5) and Δ*n*(50) are zero in Table 3. There is a small possibility of helix formation for oligomers of length ≤ 10, but longer sequences do not exist; therefore *h*(50) and *m*(50) are zero in the table. When *k*_*ann*_ > 0, increasing *k*_*ann*_ gives increased Δ*n*(5), but has little effect on Δ*n*(50), *h*(50) and *m*(50).

The fourth series in Table 3 shows that changing the helix melting rate *k*_*melt*_ has a fairly small effect on all the quantities measured. If *k*_*melt*_ = 0, every helix which forms remains in place until the end of the growth phase, and then all helices melt together. The simulation works fine in this limit. When *k*_*melt*_ > 0, short helices have some probability of melting during the growth phase, but long helices are still very unlikely to melt before the end of the growth phase, since the melting rate decreases exponentially with length (equation 5). Thus, the value of *k*_*melt*_ makes little difference to the results.

### More complex polymerization models

Above, we considered the basic model for polymerization rates in which only the correct Watson Crick base can be added opposite a template base. Here we consider the equal error-rates model defined in the Methods section, which allows non-Watson-Crick combinations with a relative error rate ε. It was observed in experiments [11,12] that rates of subsequent base addition after an initial error are very much lower. Here, we assumed that growth is completely stalled after a single mismatch. The mismatch base that was added is treated as an unpaired tail of length 1. No further addition is permitted to this tail in this growth cycle, but the same sequence may continue to grow in subsequent cycles if it is paired to a different template where the last base matches.

Table 4 shows results of simulations using the equal-error-rates model. All rate parameters are set to their standard values in Table 2. The line for ε = 0 in Table 4 is the same as the line for *k*_*add*_ = 10 in Table 3. As ε increases, sequence growth is slowed slightly because mismatches cause complete stalling. The mean and max sequence lengths decrease by a relatively small factor. The values of Δ*n* are mostly somewhat smaller for ε > 0 than for ε = 0, but the increase in length per cycle is only of order 1 when there are no errors, so the introduction of errors does not reduce this very much.

**Table 4:** Summary of quantities measured in simulations using the equal-error-rates model with four values of ε and the scaled-experimental-rates model.

	mean length	max length	*N*_*seq*_	*N*_*A*_	Δ*n*(5)	Δ*n*(50)	*h*(50)	*m*(50)
*ε* = 0	31.0	232	603	870	2.15	0.87	2.70	20.7
*ε* = 0.01	28.3	262	606	1015	2.23	1.01	2.68	20.49
*ε* = 0.05	24.2	195	727	1357	1.71	0.84	3.02	20.2
*ε* = 0.1	21.0	184	842	1277	1.21	0.60	3.46	21.09
Scaled rates, *k*_*add*_ = 10	8.6	51	1816	1584	0.3	0	0	0
Scaled rates, *k*_*add*_ = 213	26.1	224	705	1669	1.76	0.55	3.12	24.88

We also considered a rate matrix where the rates are proportional to the observed experimental rates and are different for each combination of bases (see Methods). When the scaled experimental rates are used with *k*_*add*_ = 10, there is a large reduction in the mean and max lengths compared with previous cases. This is a result of the large variance of rates in the experimental rate matrix. The smallest of the four addition rates for the WC pairs is *r*_*ex*_(*U*|*A*) = 0.204, which is much less than the mean. We therefore chose a higher value of *k*_*add*_ so the rate of the slowest WC pair was scaled to 10. This requires $k_{add}\times (\frac{0.204}{4.348})=10$, so that the mean rate of the four WC pairs is *k*_*add*_ = 213. With this higher value of *k*_*add*_, the mean and max lengths are again comparable to those for the basic rates model and the equal-error-rates model. Thus, synthesis of long strands is still possible when there is a large variance of the rates for different base combinations, provided the rate of the slowest matching base combination is fast enough.

In both the equal-error-rates model and scaled-experimental-rates model we have assumed that growth is completely stalled after an initial mismatch. In experimental measurements, the addition rate is reduced by one or two orders of magnitude after a mismatch, so it is a reasonable approximation to completely exclude further addition after a mismatch. We also considered simulations in which addition after a mismatch was still possible at a reduced rate. This leads to very little change in results relative the case of no growth after a mismatch, as would be expected, simply because these kinds of addition steps occur very rarely (results not shown).

### Can a virtual circular genome exist in our simulations?

Zhou *et al*. [22] proposed the idea of a virtual circular genome in which partially overlapping sequences were present in such a way that they could be assembled into two complementary circular strands. In that case, they argued, every sequence would have a possible template for its continued growth, and it would be possible to replicate the whole set of sequences in a kind of mutual catalysis. Although we were intrigued by this suggestion, we suspected it was more likely that the sequences in our simulation were simply diverse mixtures of random strands without any specific virtual circular sequence. Therefore, we wished to develop a method that would detect the presence of a virtual circle, should anything like this arise in our simulations.

**Fig 6:**
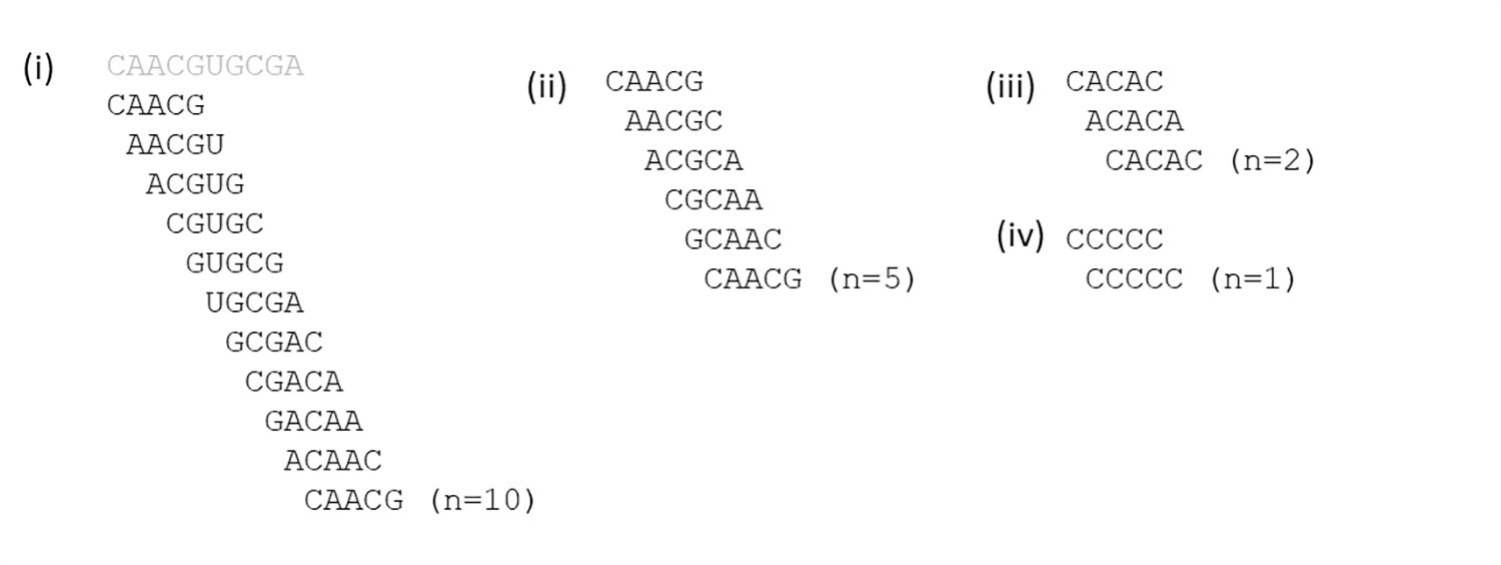
(i) A circular path of 10 steps formed from words taken from a virtual circular genome of length 10. (ii) A circular path of length 5 existing in a mixture in which all 5-mers are present. (iii) and (iv) Short circular paths arising when there is a repeating structure in the 5-mer sequence.

The three graph functions *X*(*n*), *S*(*n*) and *C*(*n*) defined in the methods section were chosen to highlight the difference between mixtures that contain virtual circles and those that do not. If there is a virtual circular genome of length 10, then there are 10 5-mers that can be formed from each strand of this genome. Fig 6i shows an example of one strand of this virtual circular genome, and a circular path of 10 words taken from this genome. Consider a perfect virtual circle sequence mixture that contains all 20 words of this genome at equal frequency and no words that are not part of the genome. In this case, all words in the mixture are part of circular paths, so *p*_*circ*_ = 1, and all the circular paths are length 10, so *C*(10) = 1. There are also circular paths for any *n* which is a multiple of 10, but not for other lengths. Thus *C*(*n*) = 0 when *n* is not a multiple of 10. For any word in this mixture there is exactly one word that can be reached by a path of length *n* steps. Therefore, the perfect virtual circle mixture has specificity *S*(*n*) = 1 for all *n*. Since there is only one word accessible by a path of length *n* from any starting word, the probability that a randomly chosen word is accessible is 1/20. Thus *X*(*n*) = 1/20, for all *n*.

Now consider a complete random mixture, where every possible 5*-*mer is present with equal frequency. In this case, it is possible to make a circular path with 5 steps from any initial word, as shown in Fig 6ii. For some words with repeating structures, circular paths shorter than 5 are possible, as in Figs 6iii and 6iv. For this complete mixture, *C*(*n*) = *X*(*n*) = 1/4^5−*n*^, for *n* < 5 and *C*(*n*) = *X*(*n*) = 1 for *n* ≥ 5. The specificity falls off with *n*: *S*(*n*) = 1/4^*n*^, for *n* < 5 and *S*(*n*) = 1/4^5^ for *n* ≥ 5.

**Fig 7:**
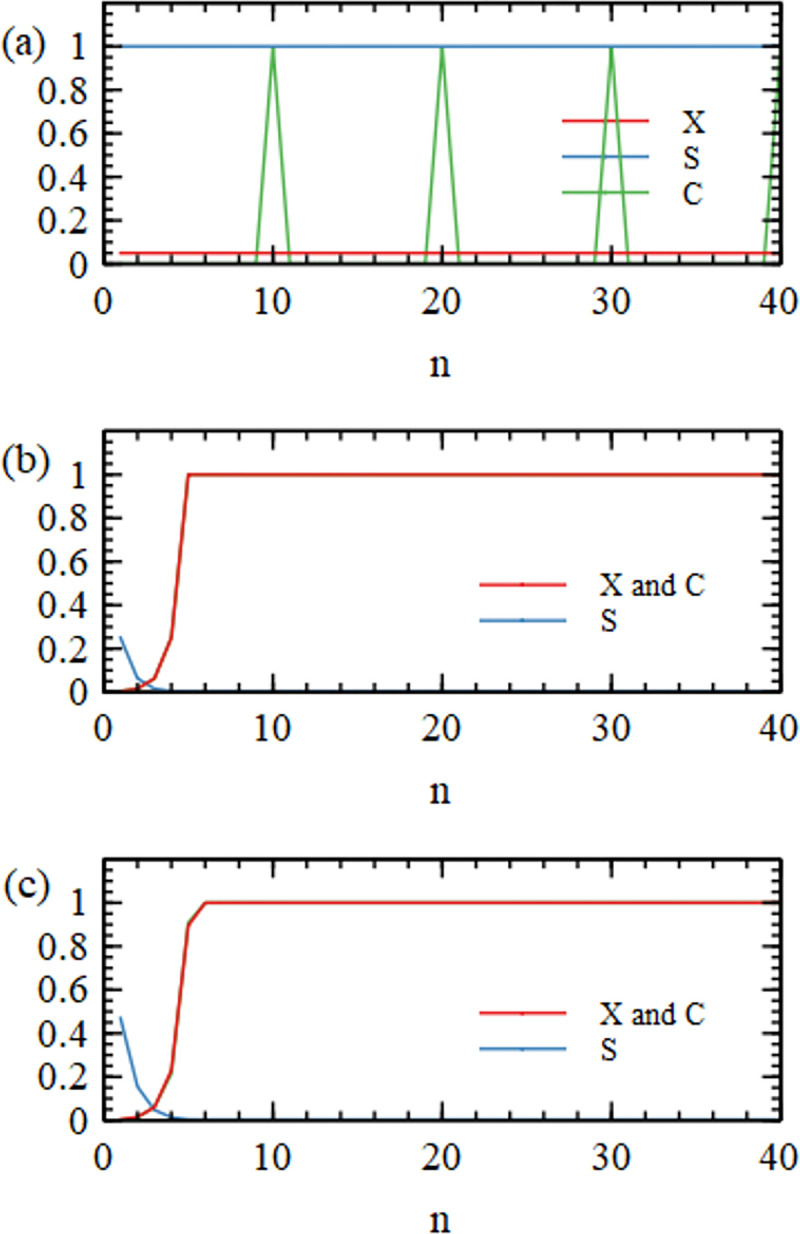
Connectivity X, Specificiy S, and Circularity C functions for (a) perfect virtual circle mixture, (b) complete random mixture with all words present, (c) simulation run 500Y1.

Fig 7A and 7B show graph functions for the limiting cases of the perfect virtual circle and the complete random mixture, in comparison to the same functions calculated for the mixture of sequences that arises in our simulation run 500Y1, shown in Fig 7C. This run uses the basic polymerization model and the standard parameter values from the earlier part of this paper. The signatures of the perfect virtual circle are that the connectivity *X*(*n*) is low but non-zero for all *n*, the specificity *S*(*n*) remains high for all *n*, and the circularity *C*(*n*) shows peaks corresponding to cycles of particular lengths. The signatures of the diverse random mixture are that *X*(*n*) increases with *n* and is 1 or close to 1 for *n* ≥ 5, *S*(*n*) is low and drops off with increasing *n*, and *C*(*n*) = *X*(*n*). The mixture in the simulation resembles the complete random mixture. We define the diversity *D* of the mixture as the number of 5-mers that are present at least once. The complete random mixture has *D* = 1024. The simulation mixture has *D =* 974. Since almost all words are present, there are very many connected circular paths in the mixture and the graph functions are similar in shape to those of the complete random mixture.

**Fig 8:**
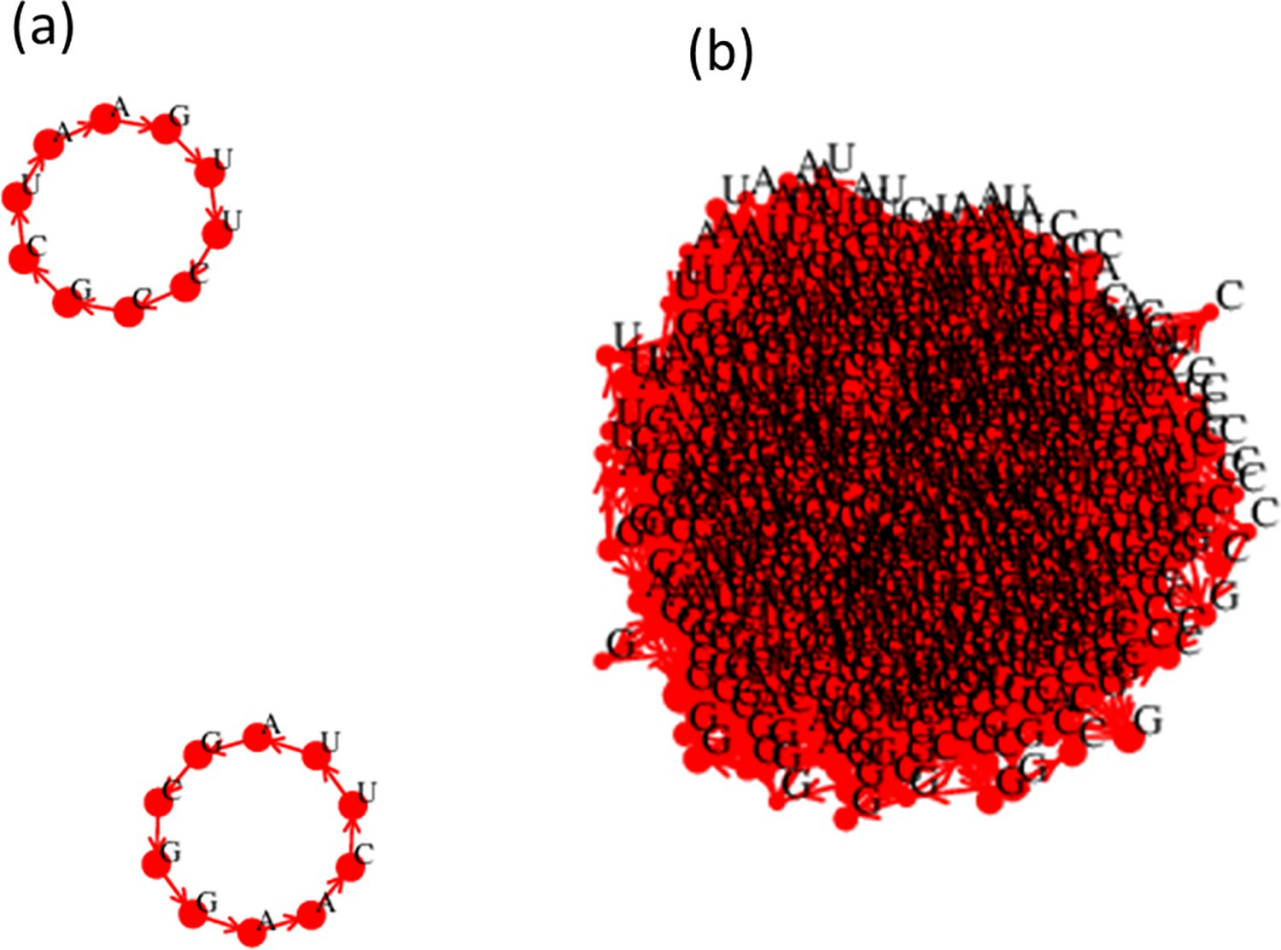
(a) Word graph from the perfect virtual circle of length 10. (b) Word graph of the diverse mixture arising in simulation 500Y1.

The word graph for the perfect virtual circle in Fig 8A consists of two disconnected circles representing the plus and minus strands of the genome. The word graph for simulation 500Y1 is a densely connected network whose structure cannot be seen in a two-dimensional plot.

From Figs 7 and 8, we see that the word diversity arising in the simulations is too diverse for a virtual circular structure to exist using the parameters we have been using so far. Word diversity depends on the initial number of random oligomers and the inflow of new random oligomers at each cycle. We therefore carried out a range of simulations intended to show the effect of deliberately limiting word diversity. The results of these simulations are summarized in Table 5. The runs are labelled in the following way. The first number indicates the initial number of oligomers, *N*_*init*_ = 500, 100, or 40. The next character indicates whether there was inflow of new random oligomers at each time step. Y indicates ‘yes’ there was inflow of *N*_*inflow*_ = *ϕN*_*init*_ new random oligomers in the usual way. N indicates there was no further inflow of oligomers other than what was in the initial set up. In both Y and N runs there is loss of sequences with a probability ϕ each time step, and there is both inflow and outflow of monomers in the usual way. The last character is either an integer, which indicates that the initial oligomers were random sequences, or C, which indicates that the system was set up with a virtual circular genome of length 10 and that all the initial oligomers were chosen from this circle or its complementary circle. All these runs were done for 1000 cycles, after which the graph functions and the word graphs were calculated.

**Table 5:** Summary of runs used to investigate the possibility of virtual circular genomes.

Run number	*N*_*inflow*_	mean length	max length	Diversity D	*p*_*circ*_	cycle type
500Y1	25	30.0	142	974	1	mixed
500YC	25	29.8	146	977	1	mixed
500N1	0	41.2	209	304	1	mixed
500NC	0	146.62	300	20	1	10
100Y1	5	38.4	283	567	0.9997	mixed
100YC	5	43.0	293	267	0.991	mixed
100N1	0	11.2	300	66	0.511	2
100N2	0	15.2	300	74	0.532	1,2,4
100N3	0	18.8	74	112	0.430	6,12,18
100N4	0	31.4	300	57	0.872	3
100N5	0	99.5	300	26	1	6,8
100N6	0	108.5	300	22	1	14,18,22
100NC	0	132.9	300	20	1	10
40Y1	2	53.4	292	181	0.998	mixed
40YC	2	68.8	300	108	0.997	mixed
40N1	0	6.0	10	58	0	none
40N2	0	6.5	10	64	0	none
40N3	0	7.6	16	54	0	none
40N4	0	8.0	142	69	0.349	6
40N5	0	20.7	300	44	0.803	4
40N6	0	58.9	265	10	1	5,10
40NC	0	136.7	300	20	1	10

The situation of no oligomer inflow in the N runs could represent a case where the random polymerization rate outside the system in negligible, or it could also represent a case where strands are inside lipid vesicles which are permeable to monomers but not oligomers. We retain the loss of strands in this case, because growth and division of vesicles would also lead to dilution and reduction of strand numbers in each cell. Later, we will explicitly model populations of protocells with growth and division, and in this later case there is neither input nor output of strands from each cell.

Run 500YC begins with a virtual circle but has continued input of new random oligomers that do not belong to the circle. The summary statistics shown in Table 5 are almost the same for 500YC as for 500Y1. Thus, there is no memory that the run began with a circular genome. The graph functions and word graph for 500YC are given in the supplementary pdf file. These are almost identical for the run 500YC as the run 500Y1. Run 500N1 begins with random sequences and has no further inflow during the run. The resulting mixture is less diverse. *D* = 304 in run 500N1 in comparison to *D* = 974 for 500Y1, but the word graph is still highly connected (see the supplement) and the shapes of the graph functions resemble those for diverse random word mixtures. Run 500NC begins with a virtual circular genome and has no further inflow. In this case, the virtual circle maintains itself. The word graph and the graph functions remain equal to those for a perfect virtual circle. The diversity remains *D* = 20, since only the words present in the initial set up can be present in the mixture. The mean and max strand lengths are much longer than the previous cases, and they are much longer than the length of the virtual circle (10). This means that long strands form that contain repeats of the whole circular sequence or the complementary sequence.

**Fig 9:**
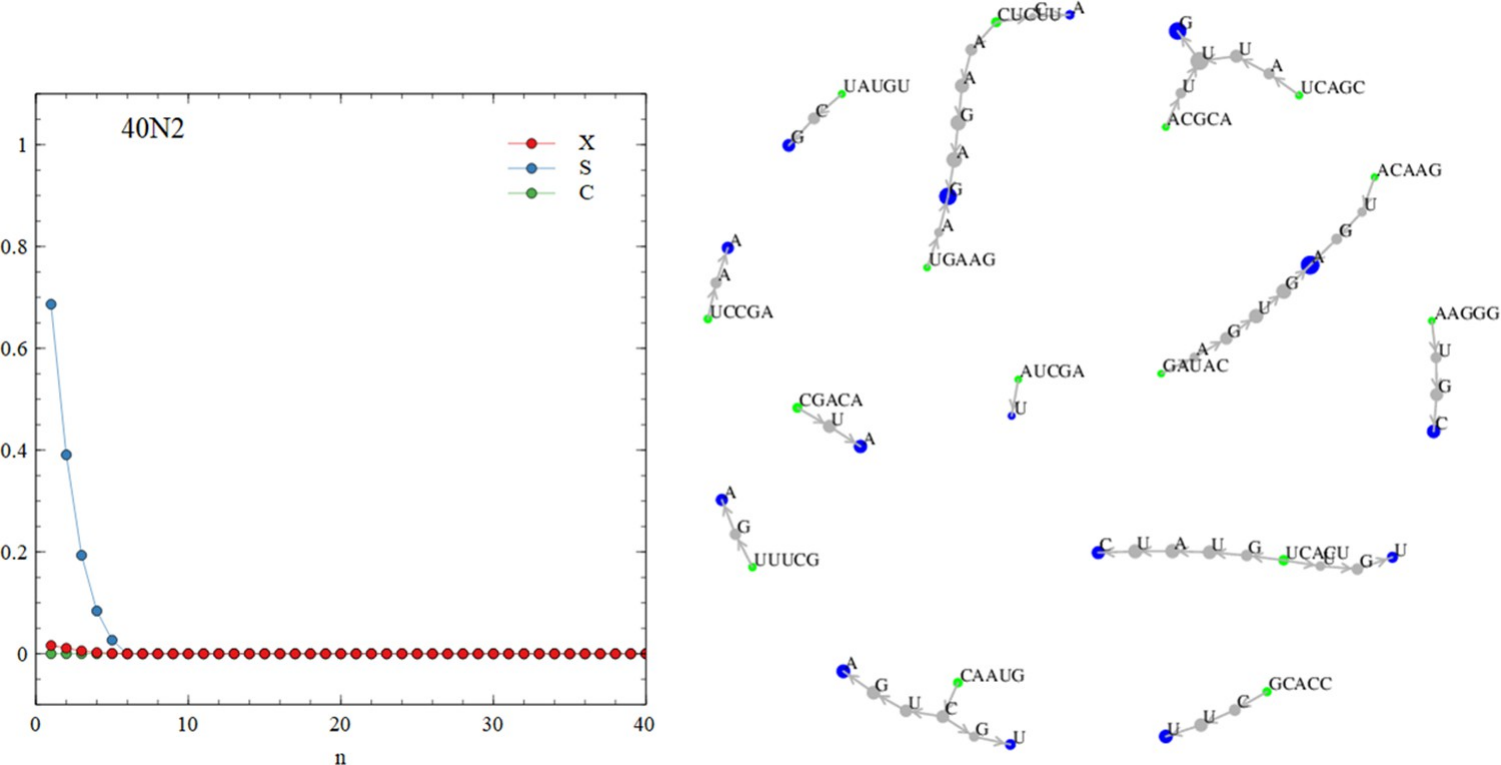
The graph functions and word graph for run 40N2 shows disconnected elements with no cycles.

**Fig 10:**
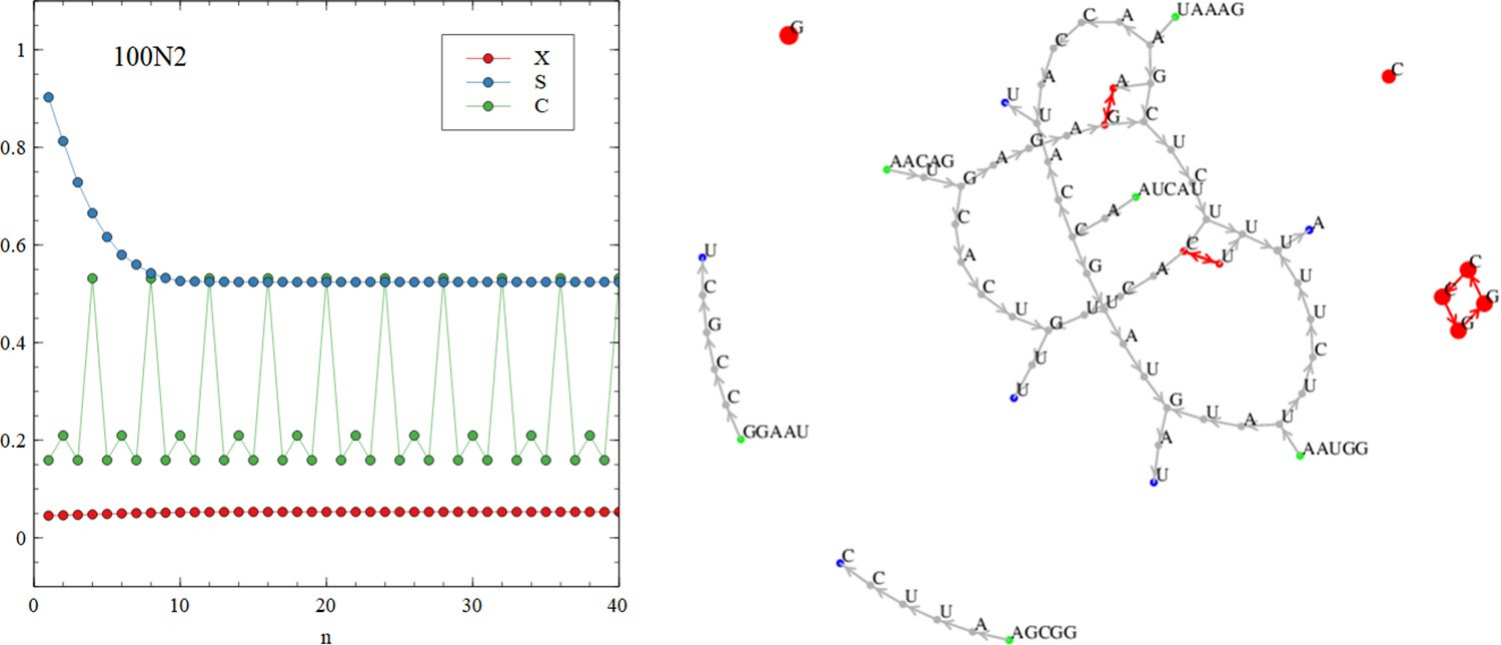
The graph functions and word graph for run 100N2 show the presence of cycles of period 1, 2 and 4.

**Fig 11:**
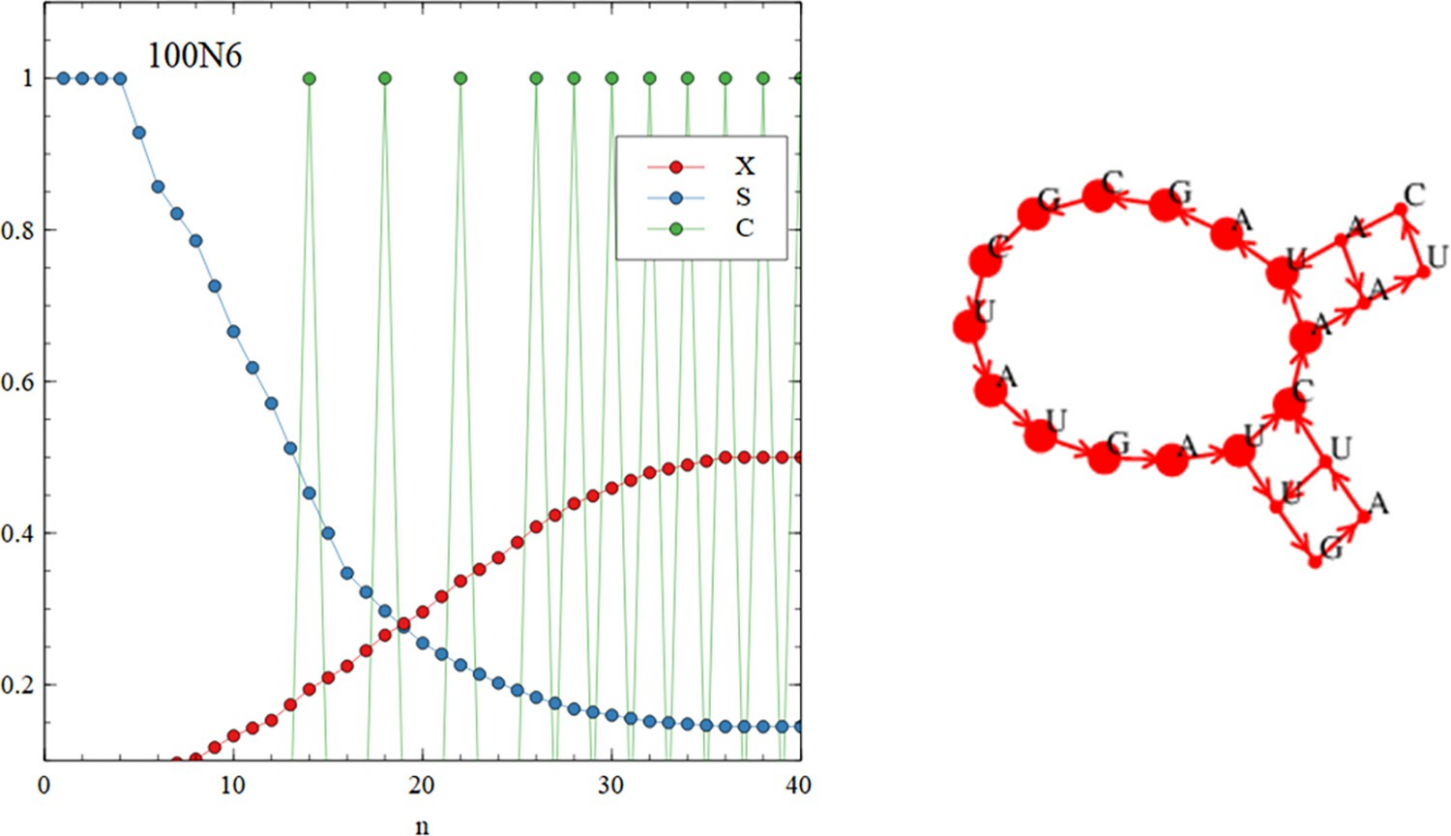
The graph functions and word graph for run 100N6 show the presence of cycles of period 14,18 and 22.

We will now consider cases where we begin with fewer sequences initially, so that the initial diversity of words is low, and where there is no inflow of new oligomers. This might arguably be the case on the inside of a small lipid vesicle. It is known that monomers, dimers and trimers can penetrate lipid vesicles [17] and potentially provide a supply for further replication, whereas longer oligomers cannot enter the vesicle. This would be similar to the ‘N’ simulations in which there is monomer inflow but no oligomer inflow. These runs are summarized in Table 5. Several examples of word graphs are shown in Figs 9–11, and the word graphs for all the runs are shown in the supplementary pdf file.

The outcomes of runs 40N1 – 40N6 and 100N1 – 100N6 are all different and depend on the particular set of random oligomers with which the simulation began. In some cases, such as 40N2 shown in Fig 9, there are no cyclic paths present in the mixture. All the words are part of non-circular paths (*p*_*circ*_ = 0), and the mean and max sequence lengths are short (see Table 5). In other cases, such as 100N2 shown in Fig 10, there is a mixture of words that are part of short circular paths, and those on non-circular paths (*p*_*circ*_ = 0.532). Fig 10 shows the present of cycles of length 1 (simple repeats CCCCC and GGGGG), length 2 (dimer repeats AGAGAG and CUCUCU) and length 4 (tetramer repeats GGCCGGCC). In cases such as 100N6, shown in Fig 11, all the words form part of cyclic paths. In this example, there is a principal cycle of length 14, and a few ‘detours’ leading to cycles of length 18 and 22 and others. The C(n) function has peaks at these values, somewhat resembling the case of the virtual circle (in Fig 7A). However, there is more than one way to travel around the word graph in Fig 11, so the mixture will contain sequences from all these possible paths and not just from one virtual circular sequence. Other examples of interesting graph structures generated by the simulations are shown in the supplementary file.

In Table 5, the runs 100N1-100N6 and 40N1-40N6 are numbered in order of increasing mean length. There is a trend that the mean sequence length is larger when *p*_*circ*_ is larger. If the original set of random oligomers happens to contain words that form cyclic paths, this permits greater extension of sequences composed of these words. If words lie only on linear paths, then the length of the sequences is limited to the length of the paths. This explains the short mean length of the sequences in runs 40N1, 40N2 and 40N3 with *p*_*circ*_ = 0, and the long mean lengths of runs 40N6, 100N5 and 100N6 with *p*_*circ*_ = 1. There is also an indication that these three runs with *p*_*circ*_ = 1 have lower word diversity than other runs that begin with the same number of oligomers. This suggests that other words that were not part of circular paths that may have been present initially in these runs have been eliminated during the simulations because they were less efficiently replicated, leaving only those words on circular paths remaining. Nevertheless, the results show many examples that contain both words on circular paths and words not on circular paths. These have survived for 1000 cycles of the simulation; thus, it is not inevitable that words that not on circular paths are eliminated in a short time.

The other runs in Table 5 confirm expectations from our runs beginning with 500 sequences. Runs 40Y1 and 100Y1 build up high word diversity and highly connected word graphs with many cyclic paths of all lengths. Runs 40YC and 100YC begin with virtual circles but lose this structure and end up resembling 40Y1 and 100Y1. Runs 40NC and 100NC begin with virtual circles and have no inflow of new words. Therefore, the virtual circular structure is maintained.

These runs show that a virtual circle does not emerge naturally in runs that begin with random oligomer mixtures. If the run is artificially set up with a mixture that only contains strands from a virtual circle, then the circle maintains itself only in an ideal case where there is no input of further oligomers with unrelated sequences. In practice, we would expect random sequence oligomers to form by random polymerization. If random polymerization did not occur at all, then it is unclear where the initial set of oligomers came from with which the simulation was initialized. Our model assumes that random polymerization outside the system gives random oligomers that can flow in, but we did not include random non-templated joining of oligomers already inside the system. If this were included, this would also lead to loss of information from the virtual circle. The simulations here are done with the basic polymerization model with no sequence errors, but if errors were incorporated, this would be a further way in which words not contained in the virtual circle would form, and this would lead to even more rapid loss of the information in the virtual circle.

### Can a functional sequence be encoded on a virtual circle?

Zhou et al [22] argued that functional RNA sequences could be encoded on the fragments of a virtual circle. We now show that this is not possible if the sequence contains repeated tetramers. Tetramer repeats are relevant here because of our earlier assumption that the minimal length of a stable helix is 4. The following argument shows what happens when a tetramer is repeated. Suppose the tetramer CCCC occurs twice in different contexts:

    . . . .ACCCCA. . . . and. . . .GCCCCG. . . .

The reverse complements must also exist:

    . . . .UGGGGU. . . . and. . . .CGGGGC. . . .

All fragments from both strands of the genome exist. Therefore, it is possible to form double helices of the form

    . . . .ACCCC

        GGGGC. . . .

Primer extension then creates

    . . . .ACCCCG and UGGGGC. . . ..

which were not in either strand of the original genome sequence. When repeated tetramers are present, words of 5 or less remain equal to those that were present in the initial genome, but words of 6 or more can be created that were not in the genome. We will now show that this leads to loss of sequence information on a length scale of 6 or more.

It should be noted that any RNA sequence that folds to a secondary structure with a stem-loop structure will contain tetramer words in stem regions that are paired with the complementary tetramers on the opposite side of the stem, for example CCCC and GGGG may be on opposite sides of a stem. If this sequence is encoded by a virtual circle, both the plus strand and the complementary minus strand of the circle will contain CCCC and GGGG. Thus, the combination of plus and minus strands is bound to contain repeated tetramers. This rules out the possibility of encoding any RNA sequence with a secondary structure (including all functional ribozymes) on a virtual circle.

The following example shows what happens if we try to encode a functional ribozyme on a virtual circle. It was shown experimentally that functional polymerase ribozymes could be assembled from fragments by using complementary fragments as splints [23]. As the plus strand of a virtual circle, we use the F ribozyme catalytic core, which is of length 100, and can be split into five fragments of length 20, as shown in Fig 3 of [23]. As the minus strand of the virtual circle we use five fragments of length 20 that act as splints. Each splint matches the final 10 bases of one fragment and the first 10 bases of the next fragment. We use five splints here, rather than the four used in [23], because we want to complete the circle.

**Fig 12:**
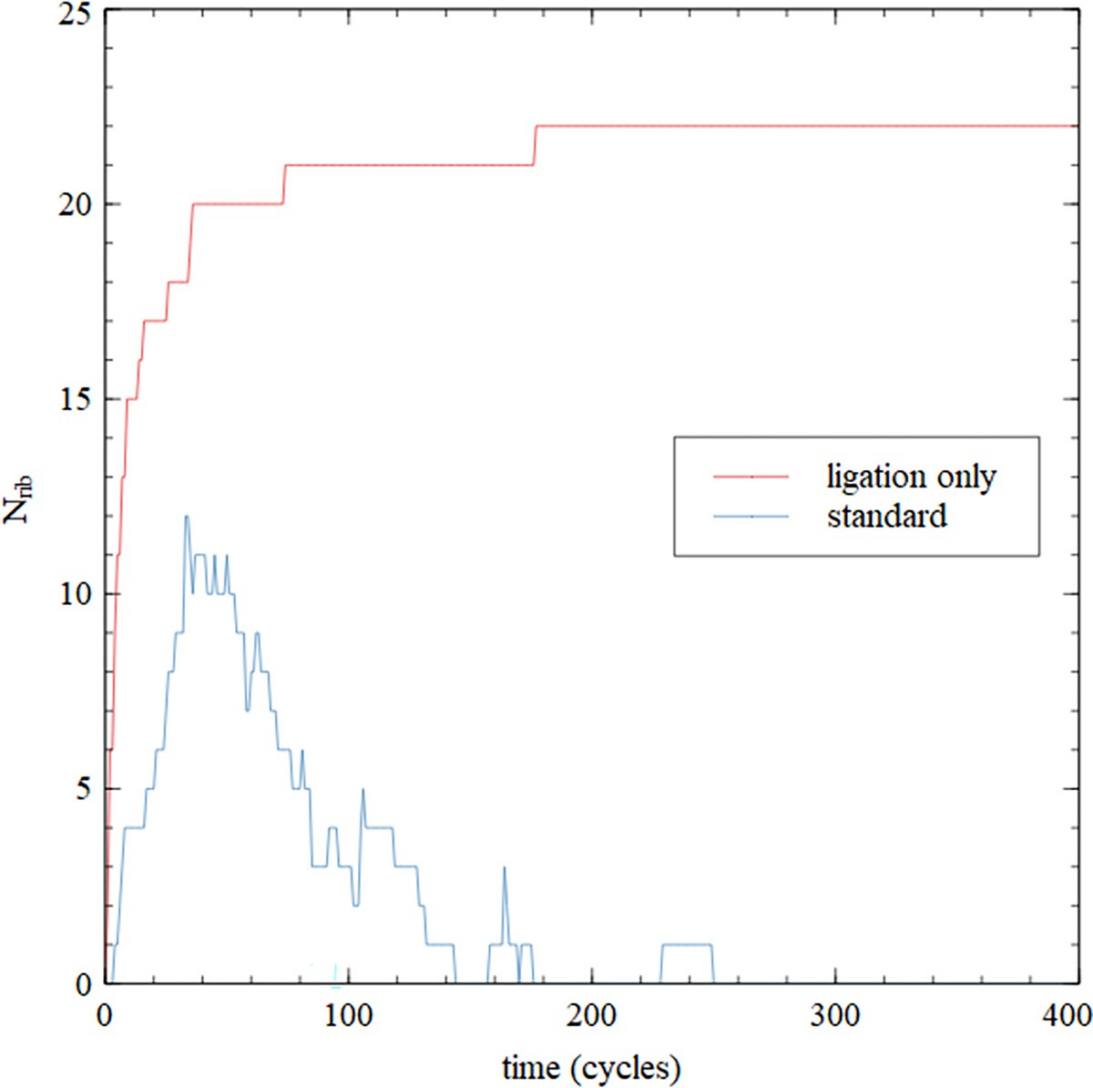
Number of complete ribozymes as a function of time for simulations beginning with the fragments and splits of the F ribozyme catalytic core. The simulation with ligation only shows that multiple copies of the complete ribozyme are assembled from fragments. The simulation with standard parameters allows monomer addition and nucleation, inflow/outflow of monomers and outflow of oligomers, as well as ligation. The full ribozyme is synthesized from the original fragments but disappears after some time because the correct fragments are not resynthesized.

The simulations in Fig 12 begin with 50 copies of each of the five ribozyme fragments and each of the five splints. Firstly we carried out a ligation-only simulation in which the monomer addition rate and the nucleation rate were set to zero. There is no inflow or outflow of any kind in this run. *N*_*rib*_ is the number of copies of the complete ribozyme sequence in the mixture, including copies which are subsequences of sequences longer than 100. Fig 12 shows that *N*_*rib*_ increases with time until all short sequences have been ligated into longer ones. There is then no further change because there is no input and output from the system. This demonstrates that the full ribozyme can be assembled from the fragments, given that the correct fragments are there initially. This is exactly what is shown by the experiment [23].

We then did a second run beginning with the same fragments using standard parameters. This run has monomer addition and nucleation in addition to ligation in the usual way. We allowed monomer inflow and outflow and strand outflow in the usual way, but no inflow of random oligomers because we do not want to introduce additional word diversity into the mixture (as with the N runs above). In this case *N*_*rib*_ increases in the early part of the run, but then reduces to zero when these copies are lost. In the later stages of the run there is a diverse mixture of sequences with a broad distribution of lengths. The sequence fragments are scrambled because there are repeated tetramers in the original virtual circle. The mixture no longer contains the correct fragments to assemble the full ribozyme; therefore no further copies of the full ribozyme can be synthesized after the early-forming ones disappear.

**Fig 13:**
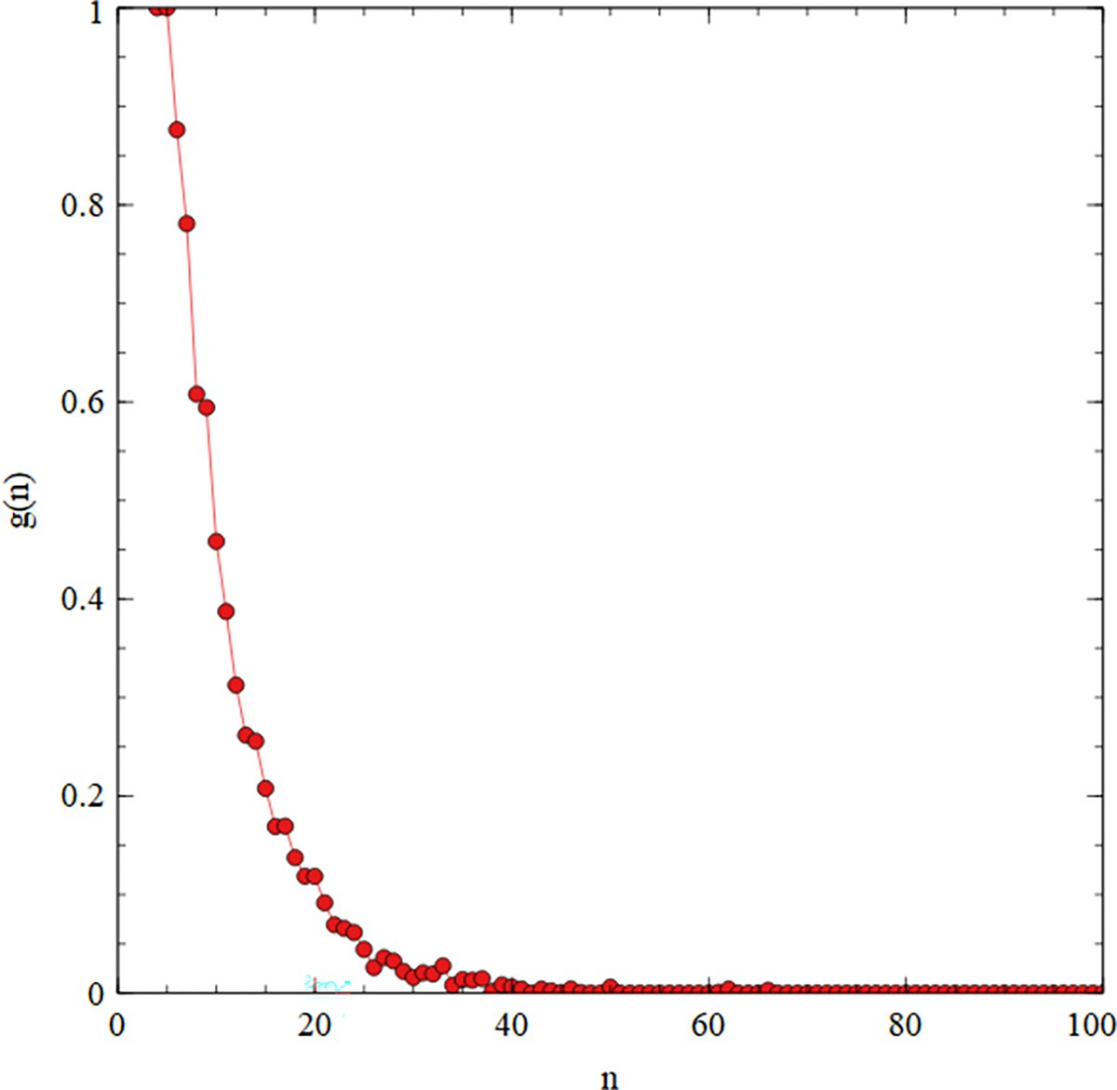
Simulation beginning with the fragments of the F ribozyme (as in Fig 12) using standard parameters. *g(n)* is the fraction of sequences of length *n* in the mixture that are still part of the ribozyme sequence or its complement after 1000 temperature cycles.

Fig 13 shows the fraction *g(n)* of sequences of length *n* that are part of the sequence of the original virtual circle (i.e. the F ribozyme or its complement). This is calculated after a long time when the sequence distribution reaches a steady state: an average is taken of cycles 500–1000. For *n* = 4 and *n =* 5, *g(n)* remains at 1 because there is no input of new oligomers. For *n* > 5, *g(n)* is less than 1 and falls rapidly with *n*. For *n =* 20, *g(n)* is only 11%, and *g(n)* is virtually zero for *n >* 50. This shows that the information on the original virtual circle is lost due to scrambling of sequence fragments. This scrambling is bound to occur whenever there are repeated tetramers, even in the ideal case where there is no oligomer inflow and no mutational error.

### Can functional sequences be maintained by selection at the cell level?

So far, we have shown that functional sequences cannot be maintained in a well-mixed set of sequences in which templating reactions are occurring with cycles of melting and reannealing. However, we have not yet considered the possibility that natural selection could help to maintain ribozymes with beneficial functions (such as polymerases, ligases, and nucleotide synthetases). It is clear that natural selection cannot operate in a single well-mixed system, because any benefit given by the ribozyme would apply to all sequences equally in the mixture, and would not increase the replication of the functional sequence relative to other random sequences. However, if there were a population of protocells, each containing its own mixture of sequences, then a beneficial ribozyme might increase the rate of growth and division of its own cell relative to others. We therefore wished to ask if it is possible that selection at the cell level could maintain a beneficial ribozyme encoded on a virtual circle, assuming that RNA synthesis occurs separately in each cell driven by cycles of melting and reannealing in the way we have been considering.

The program was modified to deal with a population of cells in the following way. Each cell has a separate mixture of sequences. Annealing, nucleation, monomer addition and ligation all occur in the usual way in each cell. Only sequences in the same cell can interact. We assume that the cell membrane is permeable to monomers, and that the concentration of monomers remains constant in each cell (rather than decreasing during the growth cycle, as was done previously). At the beginning of the simulation the total length of sequences in each cell is *L*_*init*_. Sequences are assumed to be impermeable; therefore there is no addition or removal of sequences from the cells. New sequences can be nucleated from monomers in the usual way. The simulation proceeds in time cycles as before. At the end of the growth phase of a cycle, the total length of strands in each cell is counted. If this total length ≥ 2*L*_*init*_, the cell divides. Each strand in the parent cell is independently assigned to one of the two daughter cells. Another random cell in the population is then eliminated so that the total number of cells remains constant. This introduces selection in favour of cells that grow and divide more rapidly.

We used the F ribozyme catalytic core from [23] as an example of a functional ribozyme. Each cell began with 5 complete copies of the ribozyme (length 100) and 5 copies of each of 5 fragments and 5 splints (length 20 each). The total length in each cell was therefore 1500 initially, and division occurred when the length reached 3000. There were 50 cells in the population. The number of copies of the complete ribozyme *N*_*rib*_ in each cell was tracked continually (including full copies of the complete sequence that are subsequences of longer sequences). If the ribozyme was intended to represent a polymerase, we set the monomer addition rate to be *k*_*add*_(1+*N*_*rib*_), which is different in each cell because the number of ribozymes in each cell changes during the simulation. Cells containing higher numbers of functional polymerases grow and divide faster than those with lower numbers. If the ribozyme represented a ligase, the ligation rate was set to be *k*_*lig*_(1+*N*_*rib*_). In simulations where the ribozyme has no useful function, the rates are set to their initial values *k*_*add*_ and *k*_*lig*_ independently of *N*_*rib*_.

**Fig 14:**
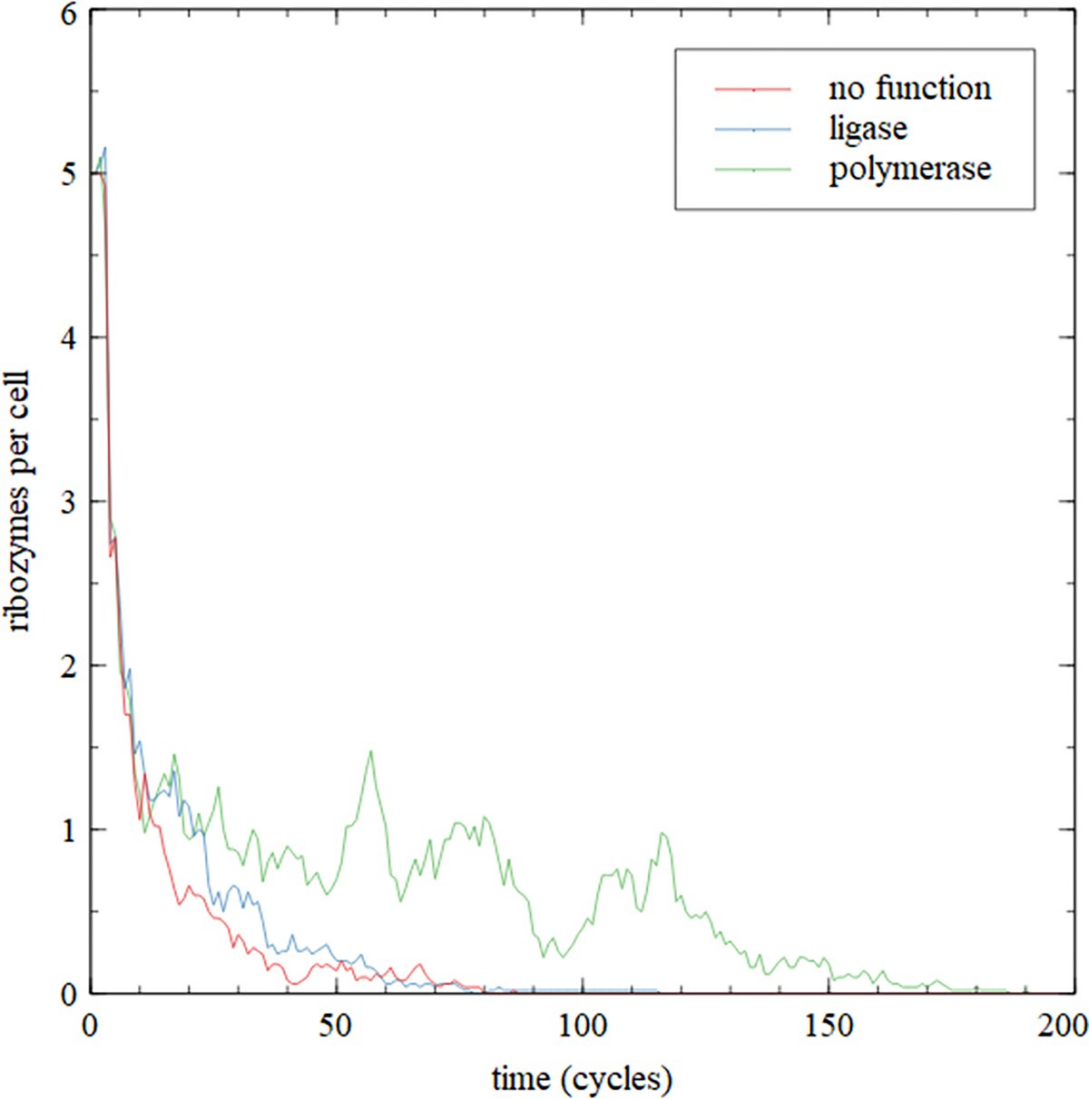
Simulations of a population of 50 cells beginning with 5 copies of the F ribozyme per cell plus fragments and splints. The ribozyme either has no function, or has a beneficial function as a ligase or a polymerase. In all cases the ribozyme is lost due to sequence scrambling.

Fig 14 shows the mean number of ribozymes per cell as a function of time. In the case of no function, *N*_*rib*_ decreases to zero in less than 100 cycles. When selection acts on ligase function, there is little difference from the case of no function. The decrease is somewhat slower when there is selection in favour of polymerase function, but the ribozymes disappear in all three cases. This suggests that sequence scrambling destroys the ribozymes, even when selection acts on a beneficial function, and even in the ideal case when there is no mutational error and no input of random oligomers.

### Maximum length of a virtual circle without repeated words

We have shown above that repeated tetramers cause sequence scrambling and prevent the maintenance of sequence information by virtual circles. It is therefore an interesting theoretical question to ask what is the maximum length of virtual circle that does not contain repeated tetramers. The number of possible tetramers is 4^4^ = 256. If these were divided into two complementary circles of length *L*, this would give a maximum possible *L* of 128. However, certain tetramers are self-complementary–they are their own reverse complement (eg. ACGU). There are 16 tetramers of this form. These cannot be included in the sequence, because if there were a self-complementary tetramer in one strand it would also be present in the complementary strand, which would violate the requirement for no repeats. The number of remaining tetramers is 256–16 = 240, which gives an upper limit of *L* = 120.

We wrote a program which searches for virtual circles without repeated tetramers. The program begins with a random sequence of length *L*. The complementary sequence is then created. The number of occurrences of tetramer *i* in both strands is *n*_*i*_. We define an energy score

$$E=\sum_{i=1}^{256}\frac{n_{i}(n_{i}-1)}{2}$$

which counts the number of repeated words. A perfect solution with no repeated words has *E* = 0. We then used a Metropolis Monte Carlo routine to minimize *E*. At each step, a new trial sequence was created by modifying the current sequence. The trial was either a single point mutation in the first strand of the genome, or a cut-and-paste change where a subsequence of a random length was deleted from the first strand of the genome and reinserted at a random point in the circle. Whenever a change was made in the first strand, the complementary change was made in the complementary strand. The trial sequence was then accepted with probability $exp(-\frac{\Delta E}{T})$. The effective temperature *T* was gradually reduced so that the sequence converged to a low-*E* state.

It was found that when *L* ≤ 114, a solution with *E* = 0 was always reached, but when *L* > 114, the best solution found always had *E* > 0 (*i*.*e*. some repeated tetramers were still present). The argument giving an upper limit of *L* = 120 is therefore insufficient. When we looked at which words were present in the *E* = 0 solution for *L* = 114, we found that self-complementary tetramers were excluded as expected, but tetramers consisting of two self-complementary pairs (eg. CGAU and CGGC) were also excluded. There are 16 such tetramers, but there are 4 of these that are also self-complementary (eq CGCG) which have already been excluded. Therefore, there are 12 additional tetramers to exclude. The number of remaining words is 256–16–12 = 228, which gives a maximum *L* of 114. The algorithm was run several times with *L* = 114 starting from a different random sequence. This gave a different sequence with *E* = 0 each time, but the same tetramers were always excluded. We are therefore confident that the maximum length of a perfect virtual circular genome without repeated tetramers is 114, although we do not have a proof of why the tetramers consisting of two self-complementary pairs must be excluded.

The above limits all arise from the assumption that the minimum helix length that is stable is *l*_*0*_ = 4. The maximum virtual circle length would be longer if *l*_*0*_ were larger. When *l*_*0*_ = 5, there is a requirement for no repeated 5-mers. The number of 5-mers is 4^5^ = 1024. There are no self complementary words for odd length words. Thus we have *L* ≤ 1024/2 = 512. Using our Monte Carlo algorithm, we were able to find a zero-energy solution for *L =* 512; therefore, we know this is the correct limit for *l*_*0*_ = 5. When *l*_*0*_ = 6, the number of 6-mers is 4^6^ = 4096, and the number of self-complementary 6-mers is 4^3^ = 64. This gives an upper limit for the length of a genome without repeated 6-mers as *L* ≤ (4096–64)/2 = 2016. Our Monte Carlo algorithm for finding sequences without repeated words proved to be very slow for *l*_*0*_ = 6. The longest length for which we have found a zero-energy solution is *L =* 1462, but the limit is probably somewhat larger than this. So, the best we can say at this point is that the limit for *l*_*0*_ = 6 lies somewhere between 1462 and 2016.

Although increasing *l*_*0*_ makes the maximum possible length *L* without repeated words longer, it does not get round the problem that any sequence that encodes a secondary structure with a stem length of at lest *l*_*0*_ must contain complementary words of length *l*_*0*_ on opposite sides of the stem, and, therefore, must contain repeated words when both plus and minus strands of a virtual circle are considered. This problem prevents encoding functional sequences on virtual circles for any *l*_*0*_. Also, increasing *l*_*0*_ makes it generally more difficult to generate long sequences. Table 6 compares several runs of the simulation with different values of *l*_*0*_ using the basic polymerization model and standard rate parameters. One point to note is that only oligomers of length at least *l*_*0*_ can grow. For this reason, we had input oligomers in the range 4–10 when *l*_*0*_ was 4. We have compensated for this, by changing the range of input oligomers to be *l*_*0*_-10. The initial number of oligomers is still *N*_*init*_ = 500, and the number of new oligomers per cycle is still *N*_*inflow*_ = 0.05 × 500 = 25 in all cases. Even after accounting for this, there is a substantial decrease in the mean and max sequence lengths as *l*_*0*_ is increased from 4 to 5 to 6. Another problem with increasing *l*_*0*_ is that we only allow annealing between two regions of strands that are exactly complementary over a window of length *l*_*0*_. Thus, the fraction of attempted annealing events that succeed in pairing decreases when *l*_*0*_ increases. In the final run in Table 6, we have increases *k*_*ann*_ (the rate of attempted annealing events) to compensate for the fact that the fraction of successful annealing events is lower. We observe that the mean and max lengths with *l*_*0*_ = 5 and *k*_*ann*_ = 100 *h*^−1^ are similar to those for *l*_*0*_ = 4 and *k*_*ann*_ = 10 *h*^−1^.

**Table 6:** Comparison of runs with different values of the minimum helix length *l*_*0*_.

	range of length of input oligomers	*k*_*ann*_ (h^-1^)	mean length	max length	*N*_*seq*_	*N*_*A*_	Δ*n*(*l*_*0*_ +1)	Δ*n*(50)	*h*(50)	*m*(50)
*l*_*0*_ = 4	4–10	10	31.0	232	603	870	2.15	0.87	2.70	20.7
*l*_*0*_ = 5	5–10	10	16.5	152	1067	1024	0.42	0.56	2.79	26.08
*l*_*0*_ = 6	6–10	10	8.6	80	1824	1731	0.048	0.00	2.67	37.44
*l*_*0*_ = 5	5–10	100	30.0	198	612	725	2.05	0.79	2.98	27.39

## Discussion

Non-enzymatic replication is a complex process whose details are not fully understood. There is still no fully-working experimental system that exhibits all the features that would be required for RNA replication at the time of the origin of life. Hence the value of computer simulations that attempt to understand how the process might work. In these simulations, we have attempted to include realistic reaction steps in a way that has not been done previously. Previous simulations have always simplified things in some way that does not seem fully realistic. For example, the templating reaction is sometimes treated as a ligation reaction of two strands that requires a matching complementary strand as a catalyst (*e*.*g*. Tupper *et al*. [24] and Tkachenko and Maslov [25]), without requiring separate steps of binding a strand separation. This ignores the problem of product inhibition, *i*.*e*. that strand separation is difficult unless there is a temperature cycling process. The stages of the cycling process are explicitly included by Roy *et al*. [28], but this work does not deal with reannealing of existing strands. It simply assumes that each strand always starts replicating from a primer that is at the 3’ end of the template. There is no ligation in this model because there is only one complementary strand bound to each template. Thus there is no blocking of polymerization by other strands bound to the template. In our simulations, we find that clusters of sequences form by reannealing, and that long strands are often connected to several other strands by separate helical regions (structures such as Fig 1C). When double stranded regions occur in the middle of sequences, there are single-stranded tails at the end of the sequences. If these tails are present, ligation of two sequences cannot occur even when the double stranded regions are on neighbouring sites of the same template. The simulations of Rosenberger *et al*. [27] allow multiple helices per template (configurations such as Fig 1B), but they do not include single stranded tails, and they do not include explicit base sequences on strands. This means that any two oligomers can always anneal (without a requirement of matching sequences), and that any two oligomers bound to the same template can always ligate (because there are no single stranded tails). The blocking effect of tails in our simulation is one reason why the mean increase in length of sequences per cycle, Δn, is relatively small, even when the monomer addition rate is high (*e*.*g*. *k*_*add*_ = 1000 in Fig 4 above).

The reannealing process is important in our results, and has both positive and negative consequences for RNA synthesis. In the simple treatment of reannealing given by Tupper et al. [18], reannealing occurs between exactly complementary plus and minus strands producing a double strand that cannot be a template. Therefore reannealing blocks replication in the simple model. In the current model, reannealing occurs in random positions between sequences that are divergent; therefore the double stranded regions typically do not cover the whole sequence. This sometimes forms configurations where monomer addition or ligation is possible. In this sense, reannealing is beneficial for RNA synthesis. The input oligomers formed by random polymerization have a maximum length of 10 in our results. Due to the presence of reannealing, sequences much longer than this can be formed. Without reannealing, sequences longer than the template cannot be formed. We did not include non-templated polymerization of sequences in the mixture in these simulations. If this were added, and if it occurred sufficiently rapidly, then long random polymers might form without the need for reannealing (as in the simulations of Roy *et al*. [28]), but it seems likely that templated primer extension would be much faster than random polymerization in a real case, as we have assumed here.

If we want to get primer binding without reannealing of long strands, then we might consider a situation where the concentration of long strands was very much less than the concentration of short oligomers and monomers. In this case random short oligomers might act as primers, or new primers might nucleate from monomers. But if short oligomers can bind to templates, then longer strands should also reanneal, unless the concentration of longer strands is extremely low. So this argument does not seem promising. Also, if primer extension starts only from very short primers without the possibility of long strands reannealing, then we have the problem that monomer addition is directional. We assumed here that monomers are added at the 3’ end of strands because the monomers have an activated 5’ group. In order to get replication beginning from short primers only, it would be necessary that the primers always bind at the end of the template (which seems like wishful thinking) or that primer extension works in both directions.

Our results show that cycles of melting and reannealing of long strands allow continued synthesis of long strands, but do not allow sequence replication. To see why no sequence information is passed on in these simulations it is sufficient to realize that each sequence is paired with many other sequences acting as templates during its lifetime. It only grows for a small number of nucleotides on any one template. Therefore, it is a combination of the sequences of all its templates and not a perfect complement of any of any one template. Sequence information is lost, except in the artificial ideal case where there is no mutational error and there is no input of new random sequences. Even in this ideal case, sequence information is lost due to scrambling whenever there are repeated short words. This prevents the encoding of long functional sequences on several shorter fragments, as we have shown with the example of the F ribozyme sequence taken from [23]. Although the fragments can correctly be ligated via the splinting mechanism to form the full ribozyme, the copying mechanism studied here does not allow continued replication of the fragments, so the functional sequences are lost. There are several other ribozymes that have mechanisms of self-assembly from fragments, including the ligase of Lincoln and Joyce [31] and the recombinase of Hayden and Lehman [32]. Neither of these could be maintained in a system like this without a separate mechanism of copying the fragments.

Most of the simulations presented here deal with a single mixture of sequences without the possibility of selection acting on functional sequences. These clearly show that sequence information is not maintained. We have briefly looked at the case of populations of cells containing separate sequence mixtures, in which case the possibility of maintaining sequences by selection at the cell level exists in theory. But our results show that scrambling of sequence fragments still leads to loss of the functional sequences even when selection benefits cells containing full ribozyme sequences, and even if the mutational error rate is zero. If, on the other hand, there were a mechanism of copying each of the fragments completely from one end to the other in one cycle, then the correct fragments would be replicated, and selection at the cell level would maintain cells containing the full ribozyme sequences. The problem is that melting and random reannealing does not correctly replicate the fragments.

The suggestion that sequence information could be encoded in a virtual circular genome [22] is intriguing, and we have tried hard to find conditions where it might work. We investigated the possibility that structures like the virtual circle might spontaneously arise in mixtures where the diversity of words was low. When we began with a smaller number of initial oligomers (either 100 or 40, rather than 500), and when no further input of random oligomers occurred, then sometimes these mixtures contained words that are connected via circular paths. The word graphs of cases such as this are shown in Figs 9, 10 and 11 and further examples are in the supplementary file. It should be noted that cyclic paths do not always emerge in the low-diversity mixtures, as in Fig 9 and runs 40N1, 40N2, and 40N3 in the supplement, and that sequences that are not part of circular paths are still maintained over large numbers of cycles. Where cyclic structures did emerge in the simulations (all the runs with *p*_*circ*_>0 listed in Table 5), the lengths of these cycles were usually short, and often cycles of more that one length existed in the mixture. It would be very unusual for a combination of words to exist in a mixture that could form a long cycle, because if word diversity is low, long connected cycles will not exist, and if word diversity is high, many alternative cyclic paths will exist, most of which will be very short (5 steps or less).

Given all these difficulties associated with non-enzymatic replication, how could replication actually have got started in the RNA World? The arguments given by Tupper and Higgs [18] in favour of rolling circle replication still apply. We argued that using temperature cycling as a means to avoid product inhibition is ineffective, because reannealing of existing strands occurs much more rapidly than synthesis of new ones. The current results modify this by showing that continued synthesis of new random sequences driven by temperature cycling is possible, but that no replication of sequence information is possible. This suggests that a mechanism requiring strand displacement is preferable. Strand displacement driven by a polymerase protein occurs in the replication of viruses and viroids. In contrast, there are no naturally occurring sequences that replicate via a temperature cycling mechanism. A strand displacement model for RNA replicators has also been studied by Takeuchi *et al*. [33].

We also pointed out [18] a problem for strand displacement on a linear strand. If a partially-complete new strand is displacing a long complete complementary strand, then it is likely that the growing 3’ end of the new strand will separate from the template and that branch migration will occur, leading to the loss of the incomplete new strand from the template before reaching the end of the sequence. The old complete strand is almost bound to win this competition, since if the branch point migrates to the beginning of the template, the short incomplete strand is lost, whereas if the branch moves the other way, the long sequence cannot be lost unless the synthesis of the new strand continues to completion. This problem is eliminated if the template strand is circular, and the rolling circle mechanism is occurring. In this case, the growing 3’ end is displacing the other end of its own strand, and the complementary strand cannot be completely lost from the template. We emphasize that the rolling circle mechanism should not be confused with the virtual circular genome proposal discussed in the present paper. The former involves strands that are covalently linked to form a circle; the latter involves linear strands whose sequences can be arranged in a circular fashion.

The RNA World theory remains compelling as a general scenario for the origin of life. Replication of nucleic acid sequences must have evolved at some stage, even if one does not subscribe to an origins theory in which RNA replication is primary. Template-directed replication is a surprisingly complex process. We are gradually making progress in understanding the theoretical details of the primer extension process and in getting it to work in the laboratory, but important puzzles still remain, as we have seen here.

## Supporting information

S1 Fig Contains Figures S1.01 –S1.22. Each figure corresponds to one of the simulation runs in Table 5. For each run, the plot of the graph functions X(n), S(n) and C(n) is shown, together with the word graph. Parameters for each of the runs are described in the text in the section "Can a Virtual Circular Genome exist in our simulations?".
(PDF)
