# Supplementary material for: Computer simulations of Template-Directed RNA Synthesis driven by temperature cycling in diverse sequence mixtures
Source: PLoS Comput Biol. 2022 Aug 24;18(8):e1010458. doi: 10.1371/journal.pcbi.1010458 (PMC9447872; doi:10.1371/journal.pcbi.1010458)

Fig S1.01: 500Y1

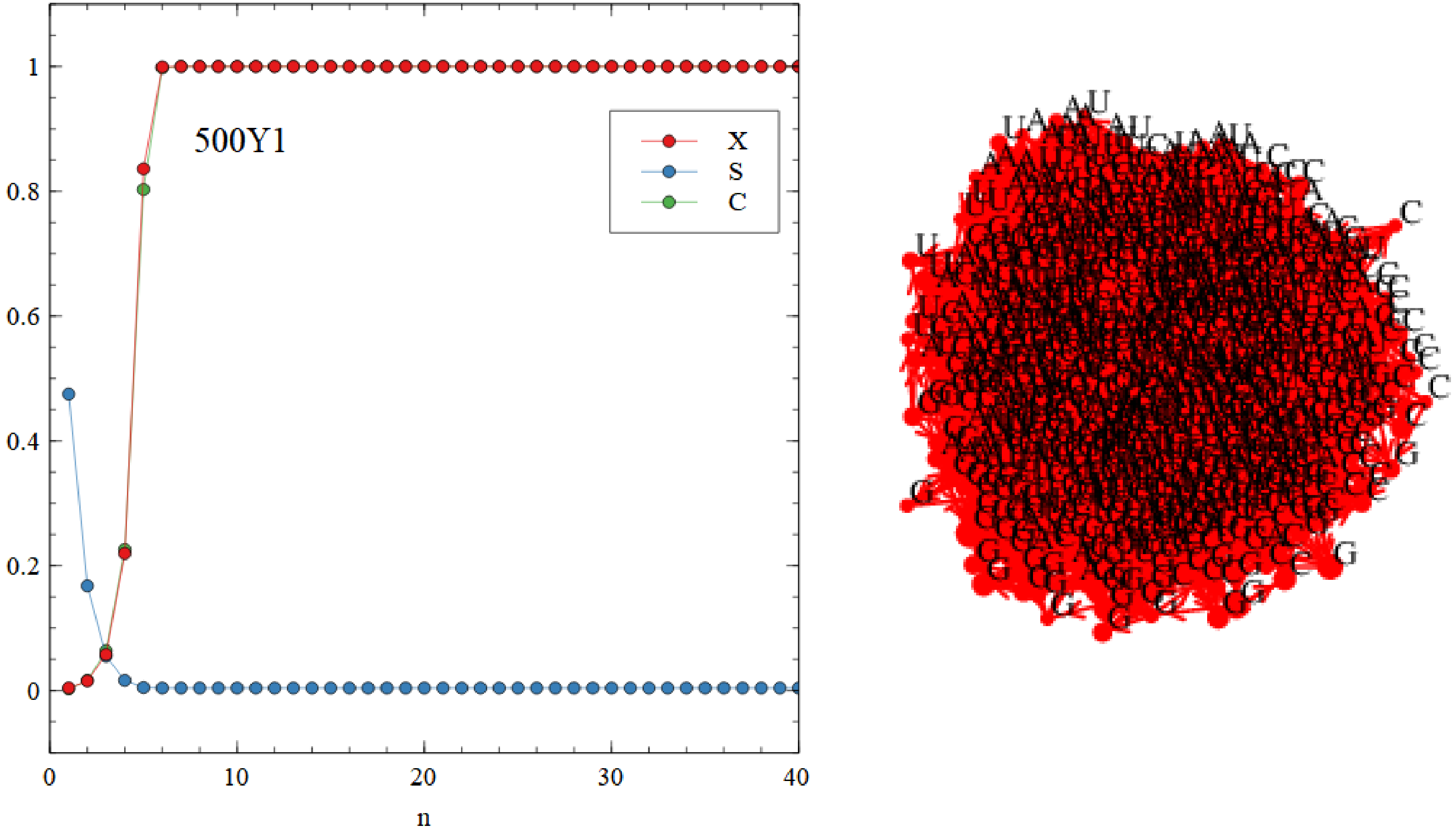

Fig S1.02: 500YC

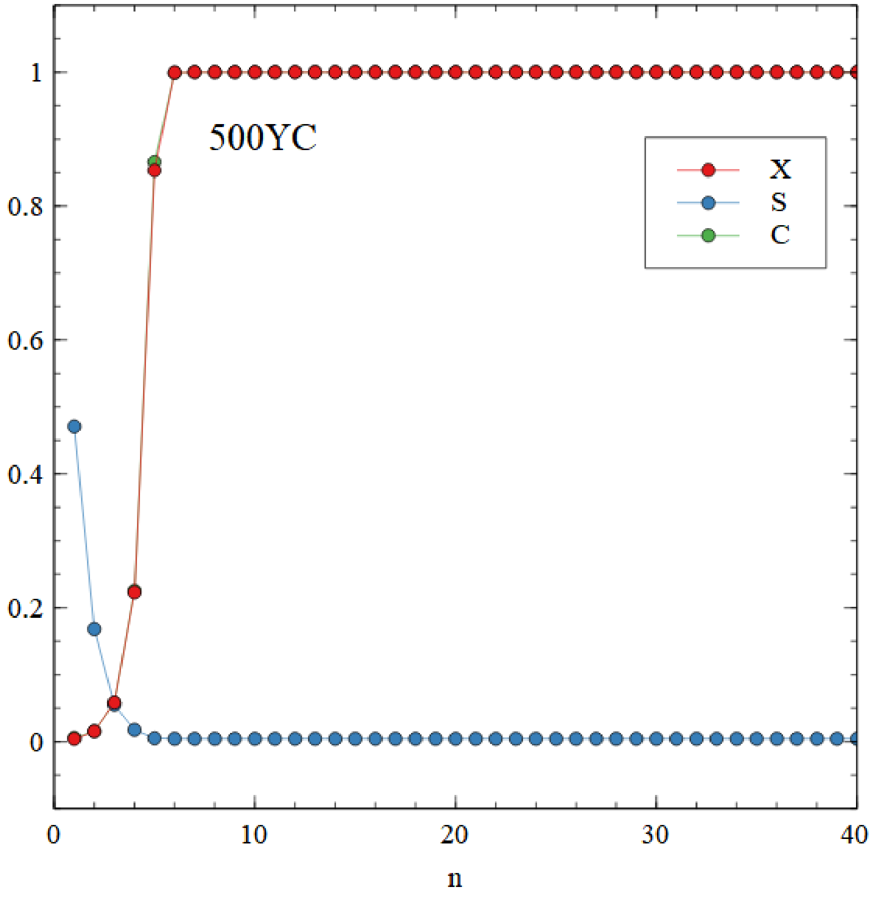

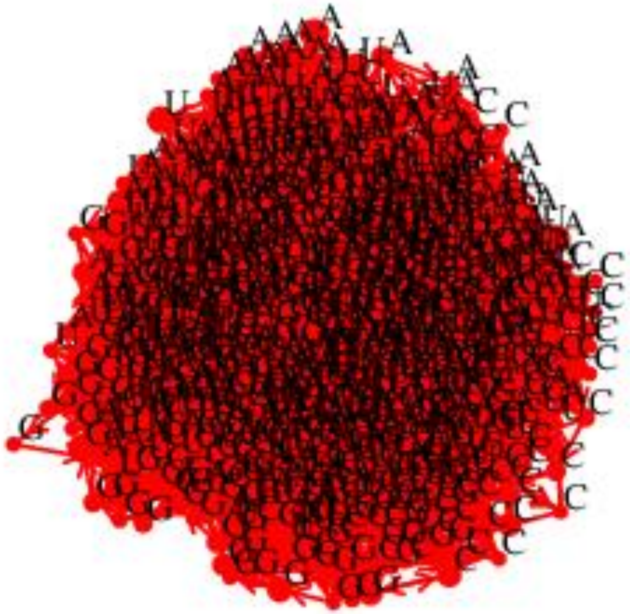

Fig S1.03: 500N1

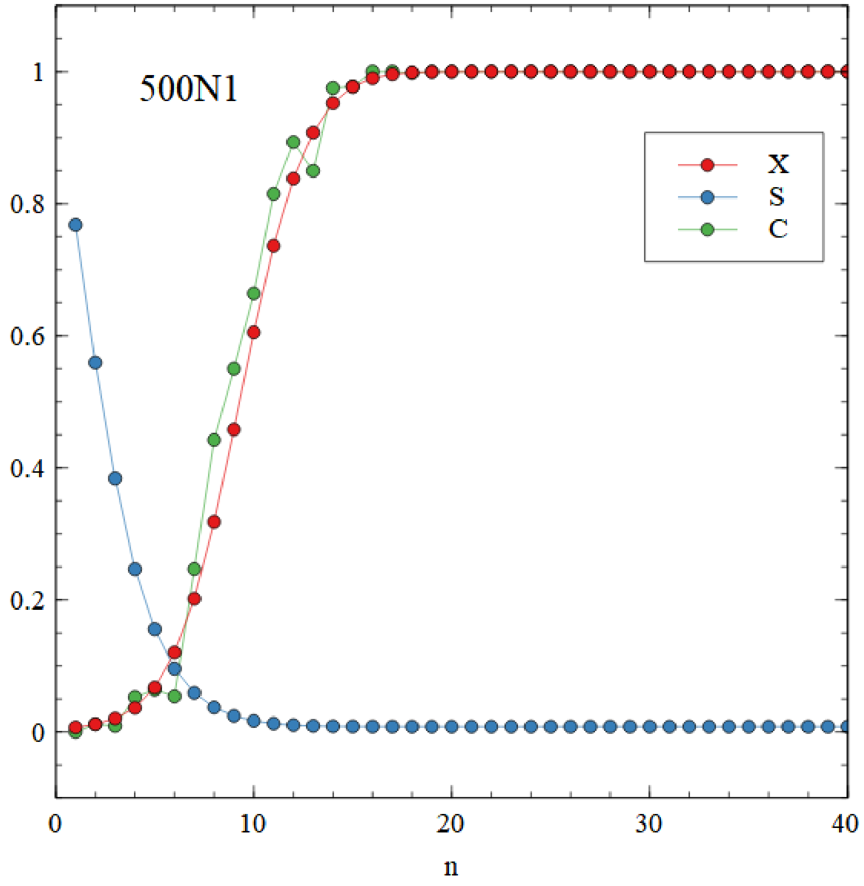

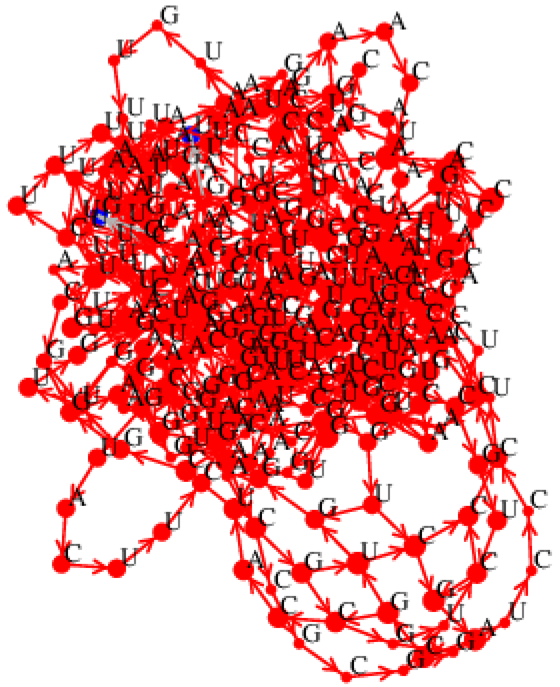

Fig S1.04: 500NC

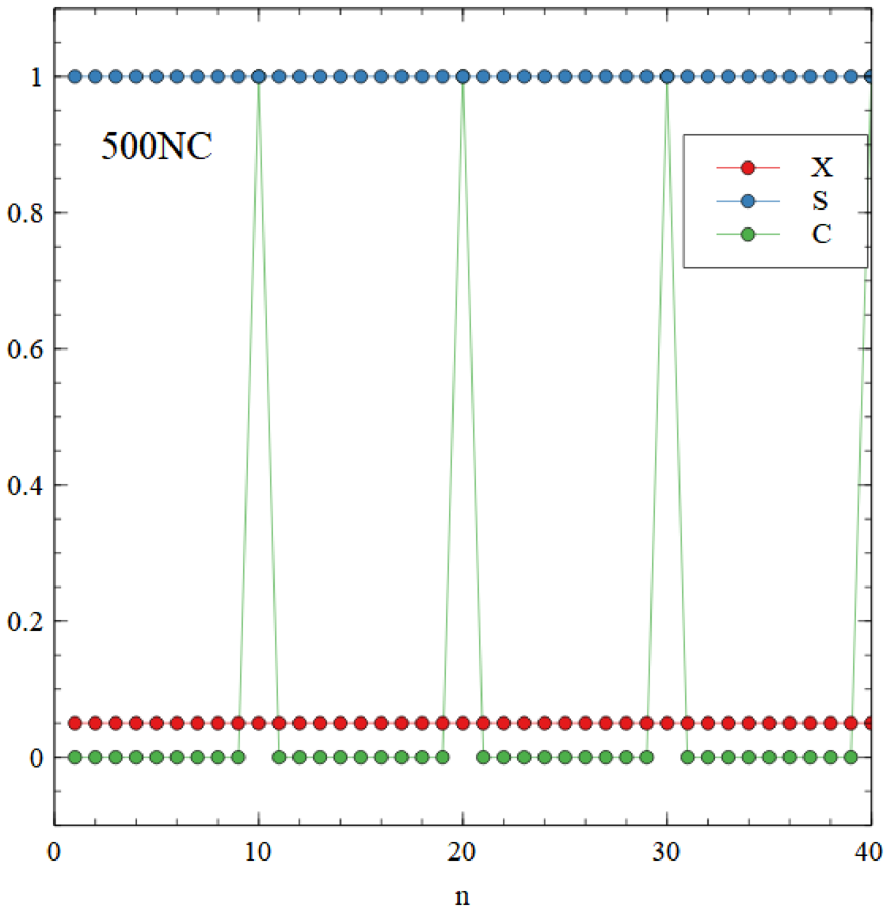

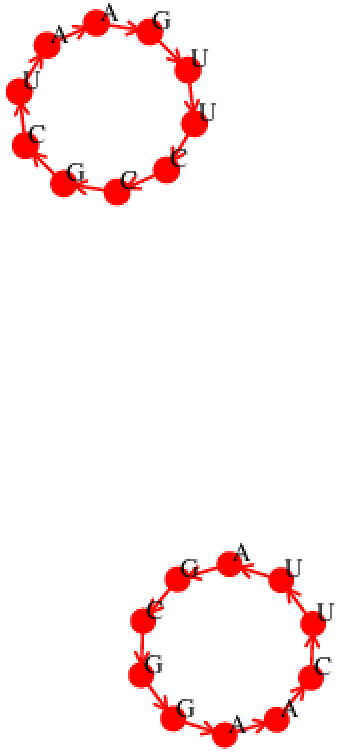

Fig S1.05: 100Y1

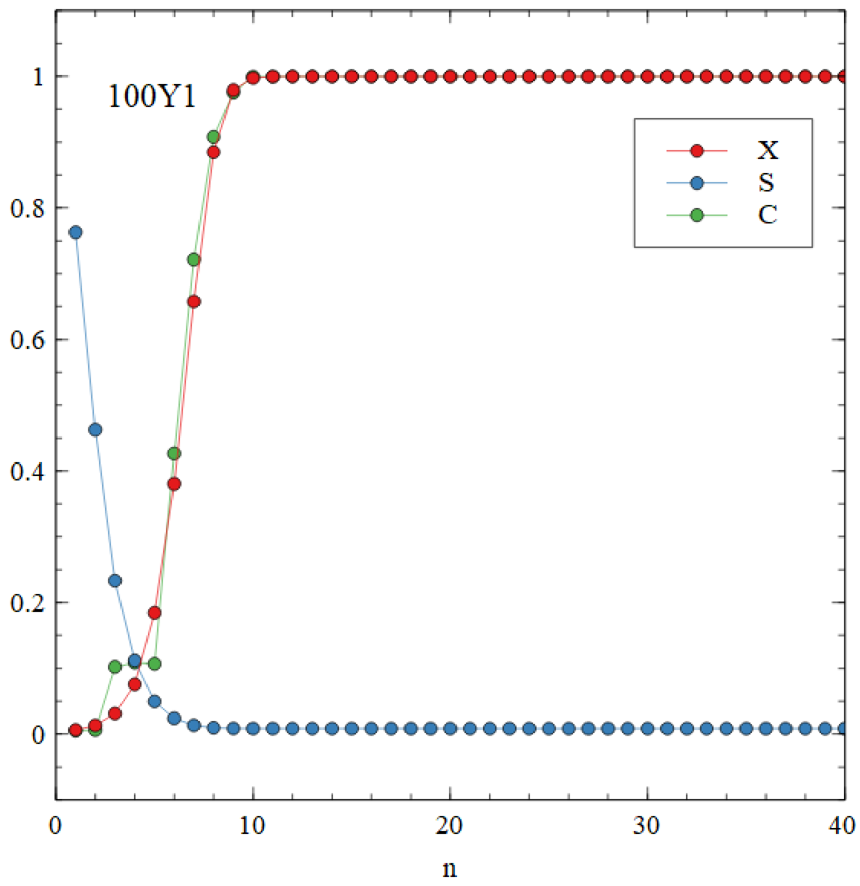

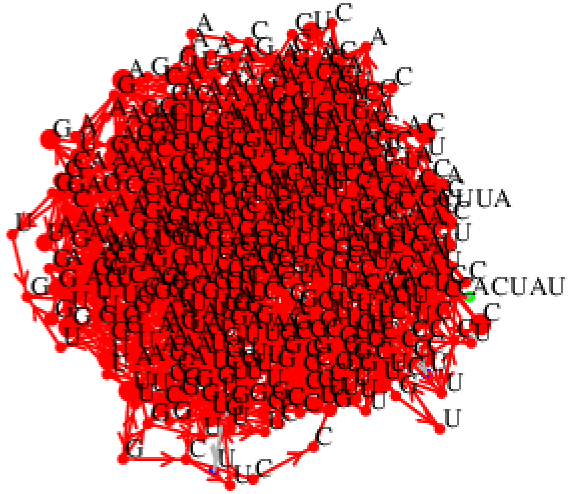

Fig S1.06: 100YC

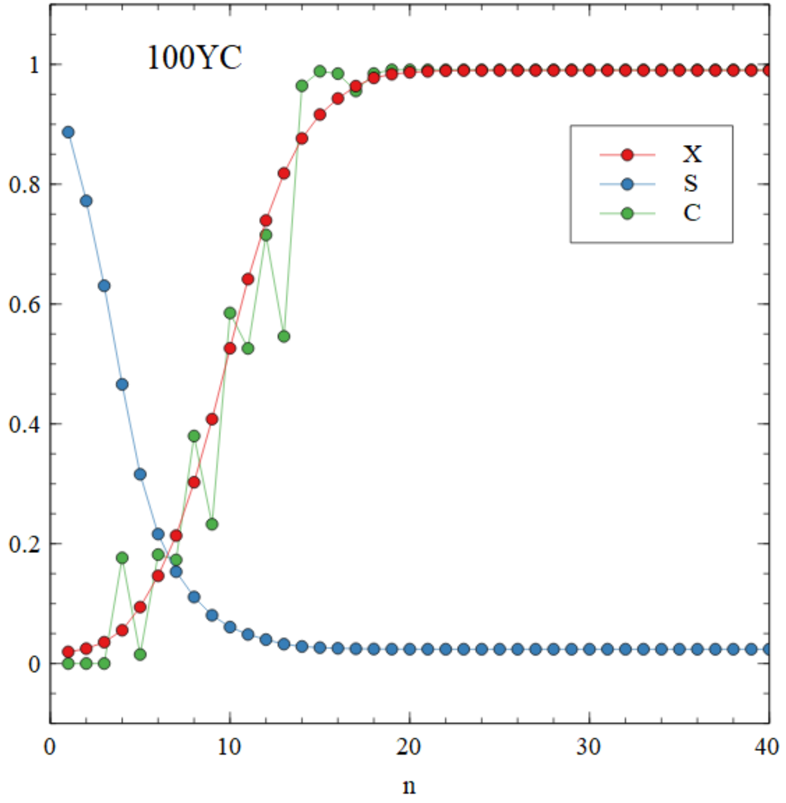

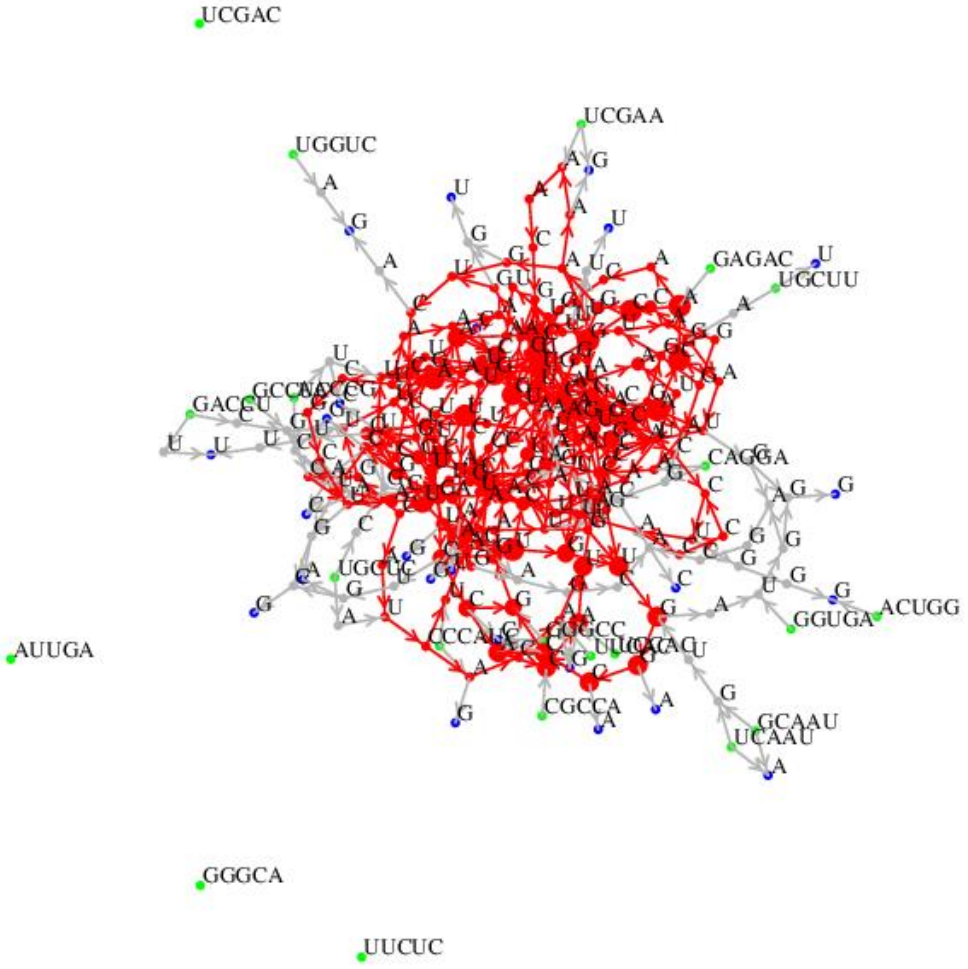

Fig S1.07: 100N1

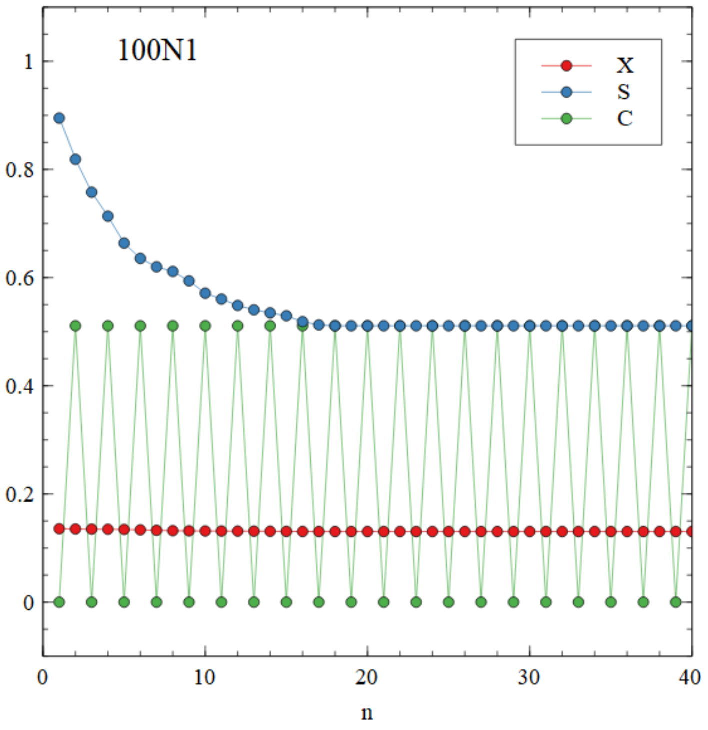

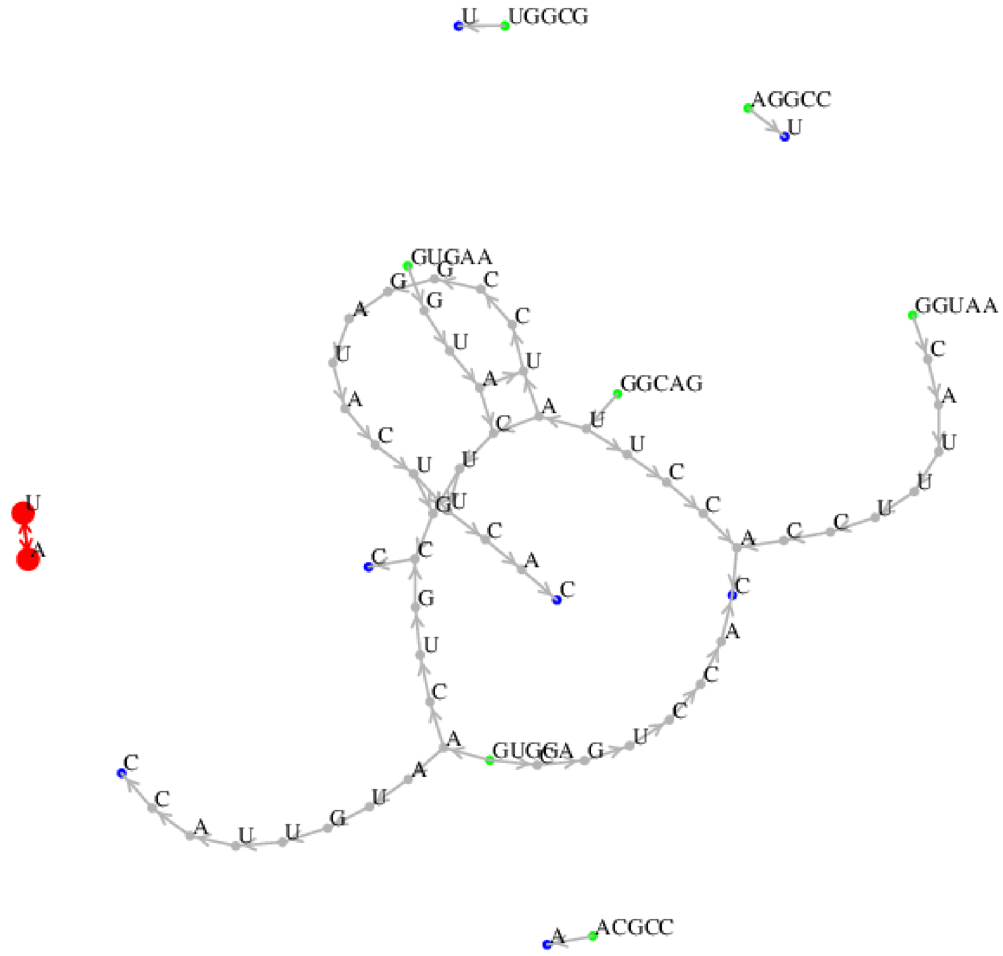

Fig S1.08: 100N2

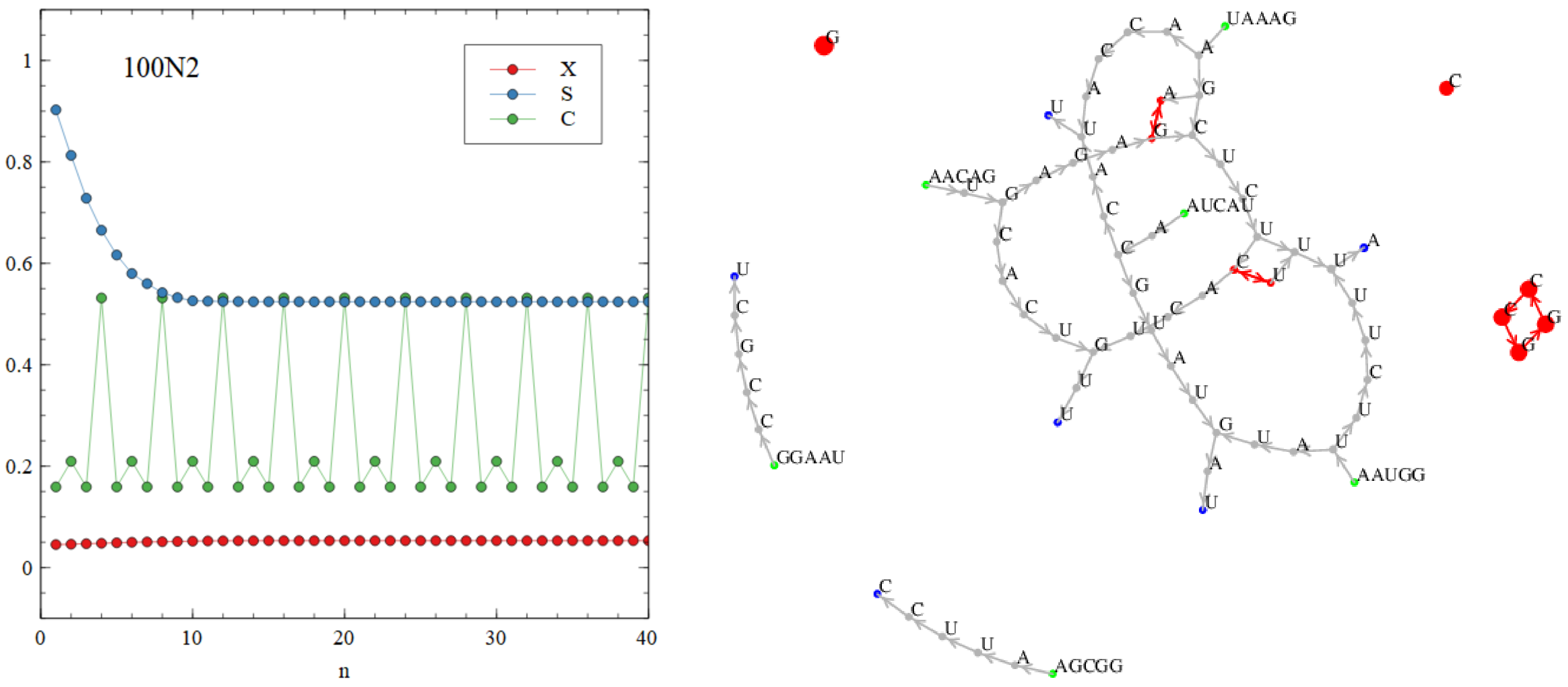

Fig S1.09: 100N3

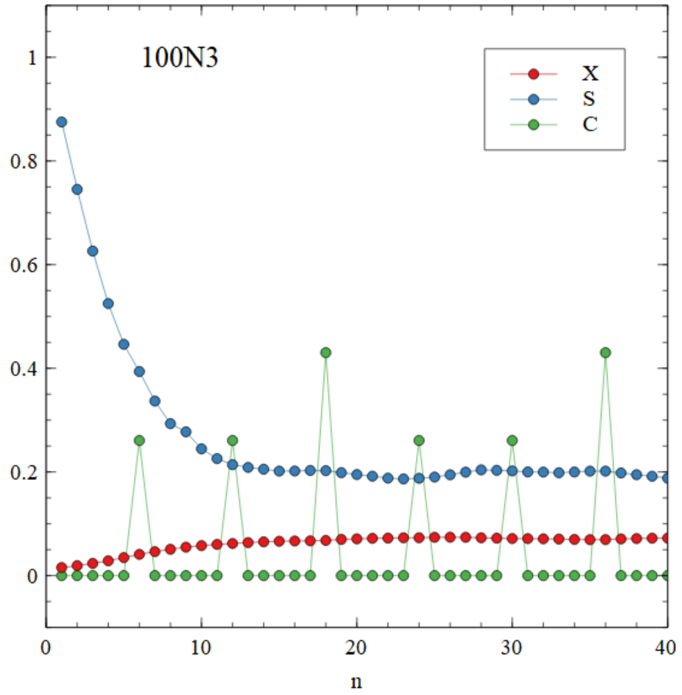

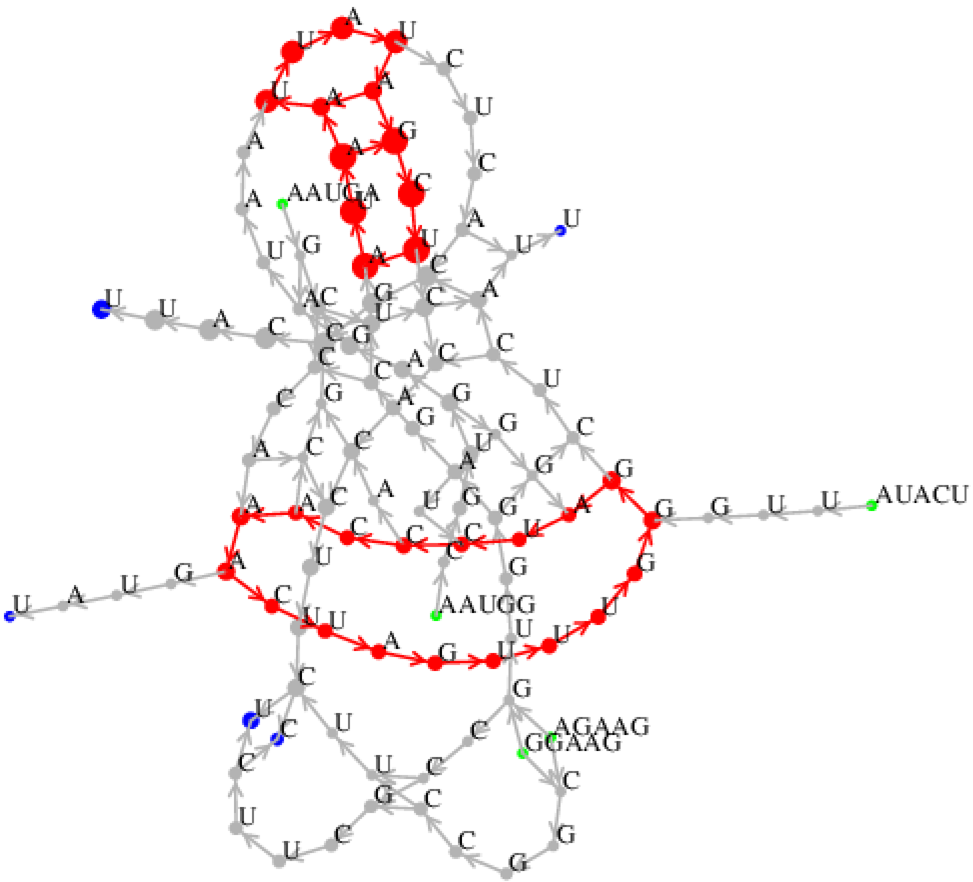

Fig S1.10: 100N4

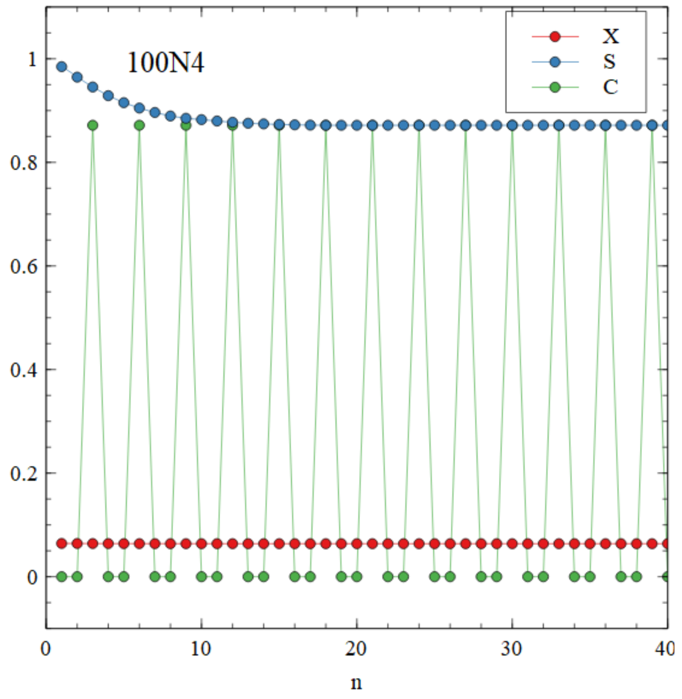

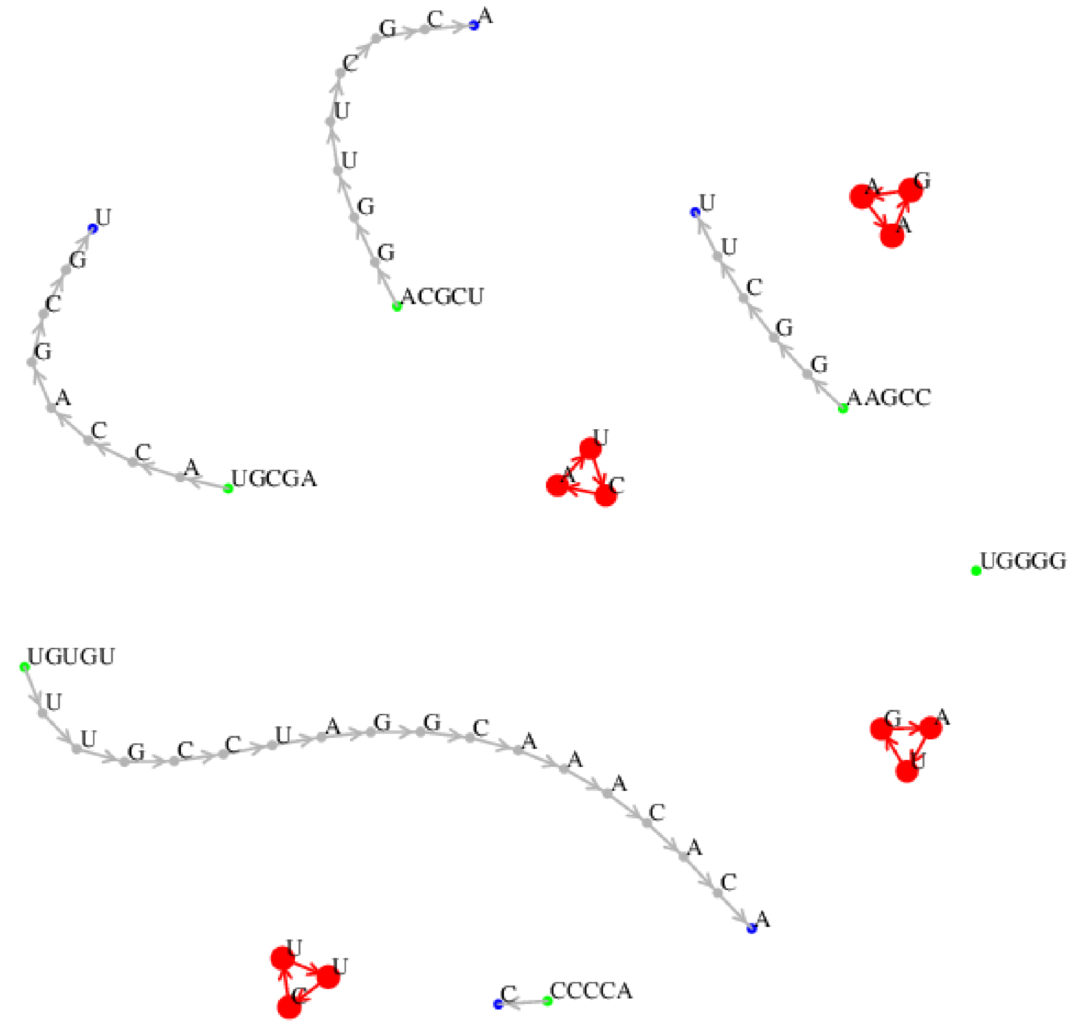

Fig S1.11: 100N5

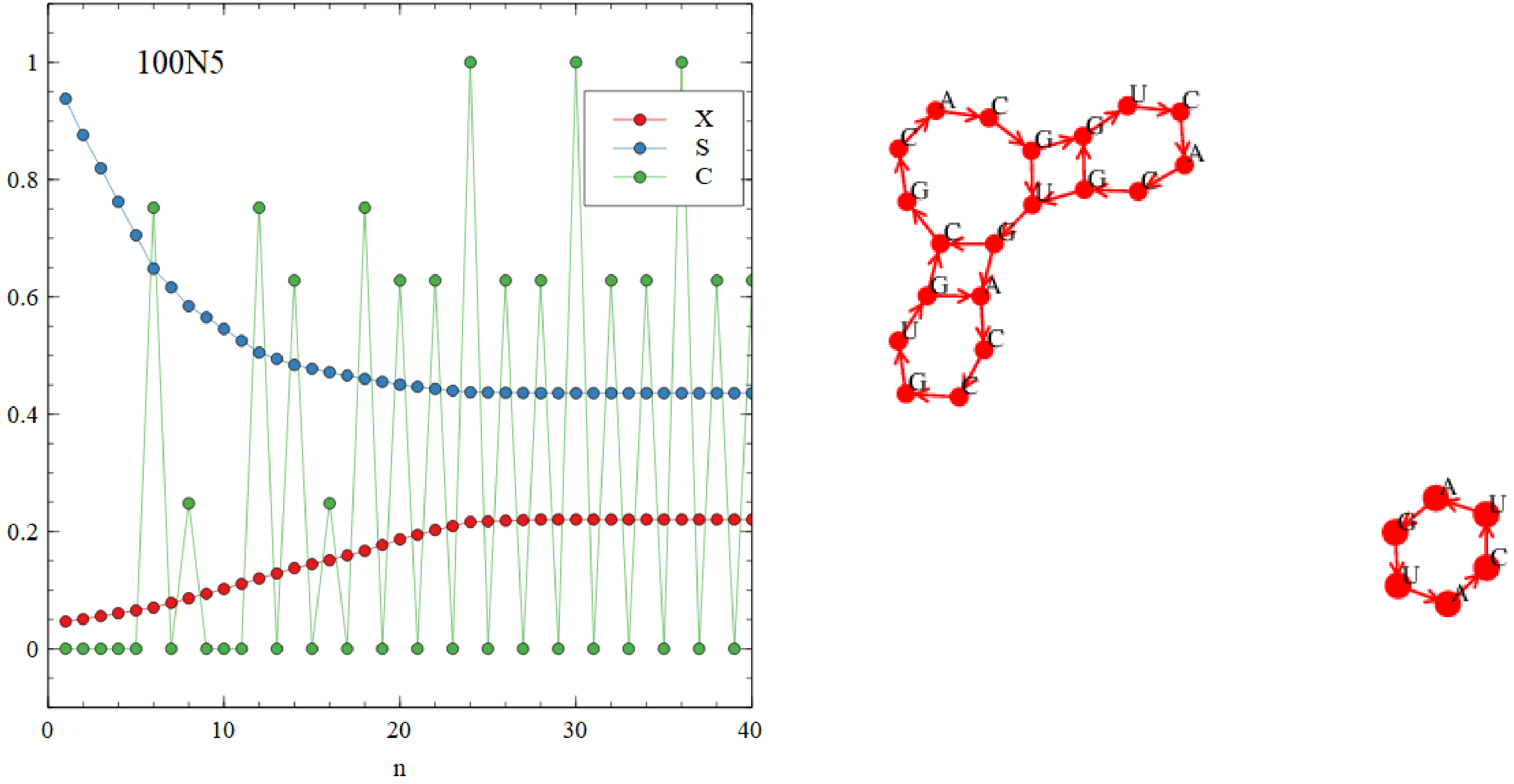

Fig S1.12: 100N6

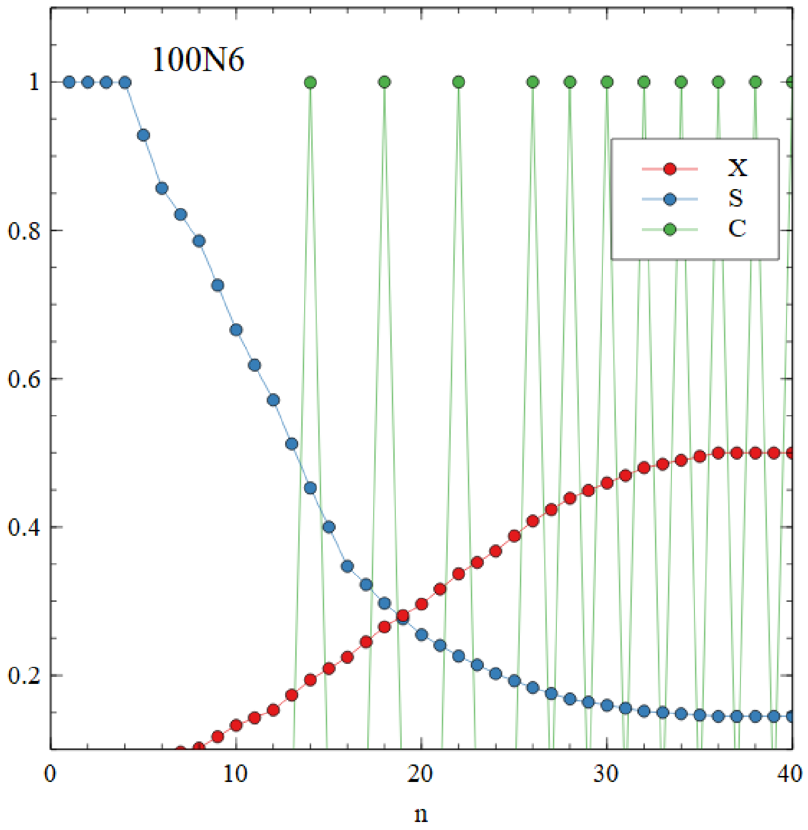

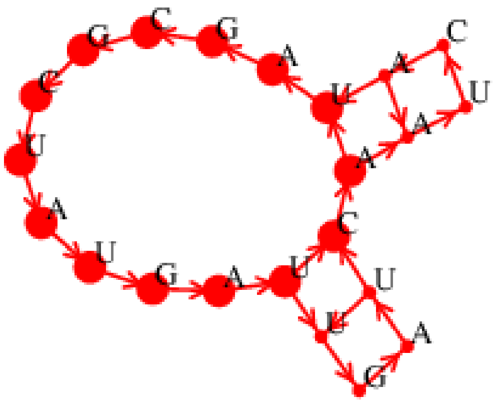

Fig S1.13: 100NC

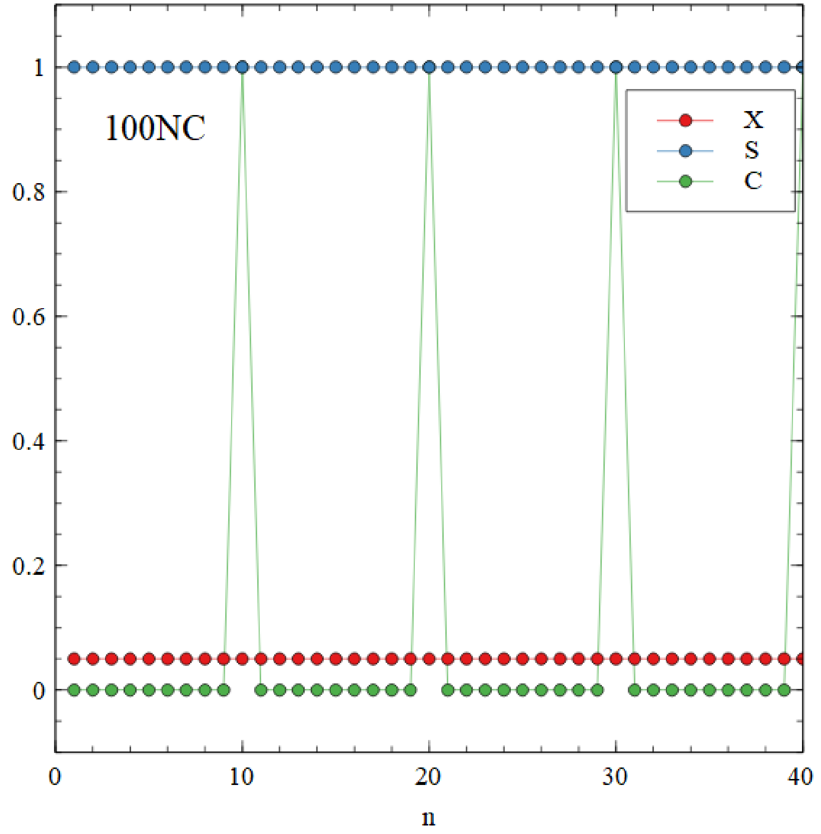

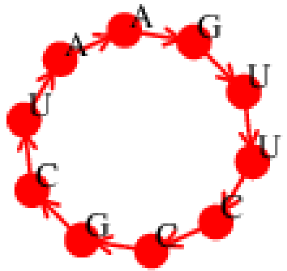

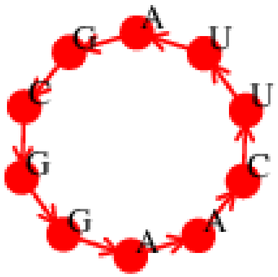

Fig S1.14: 40Y1

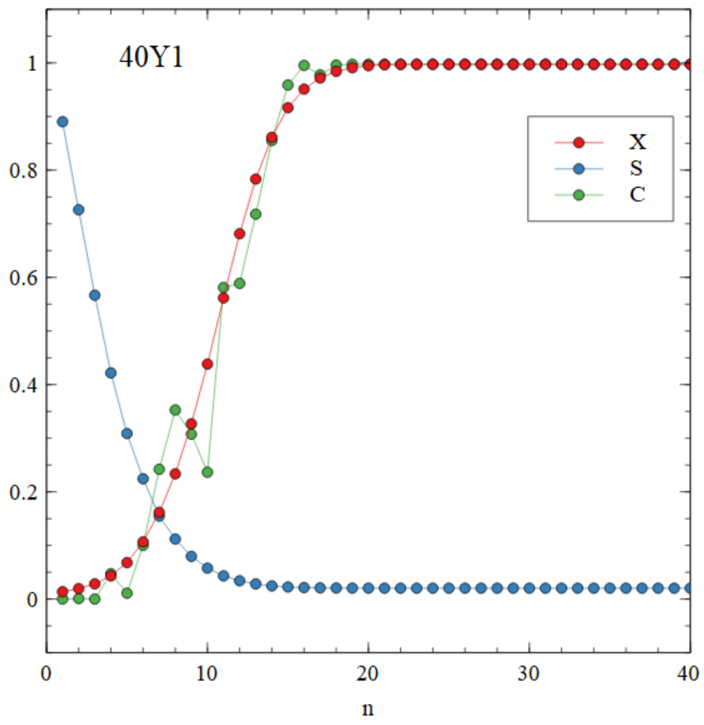

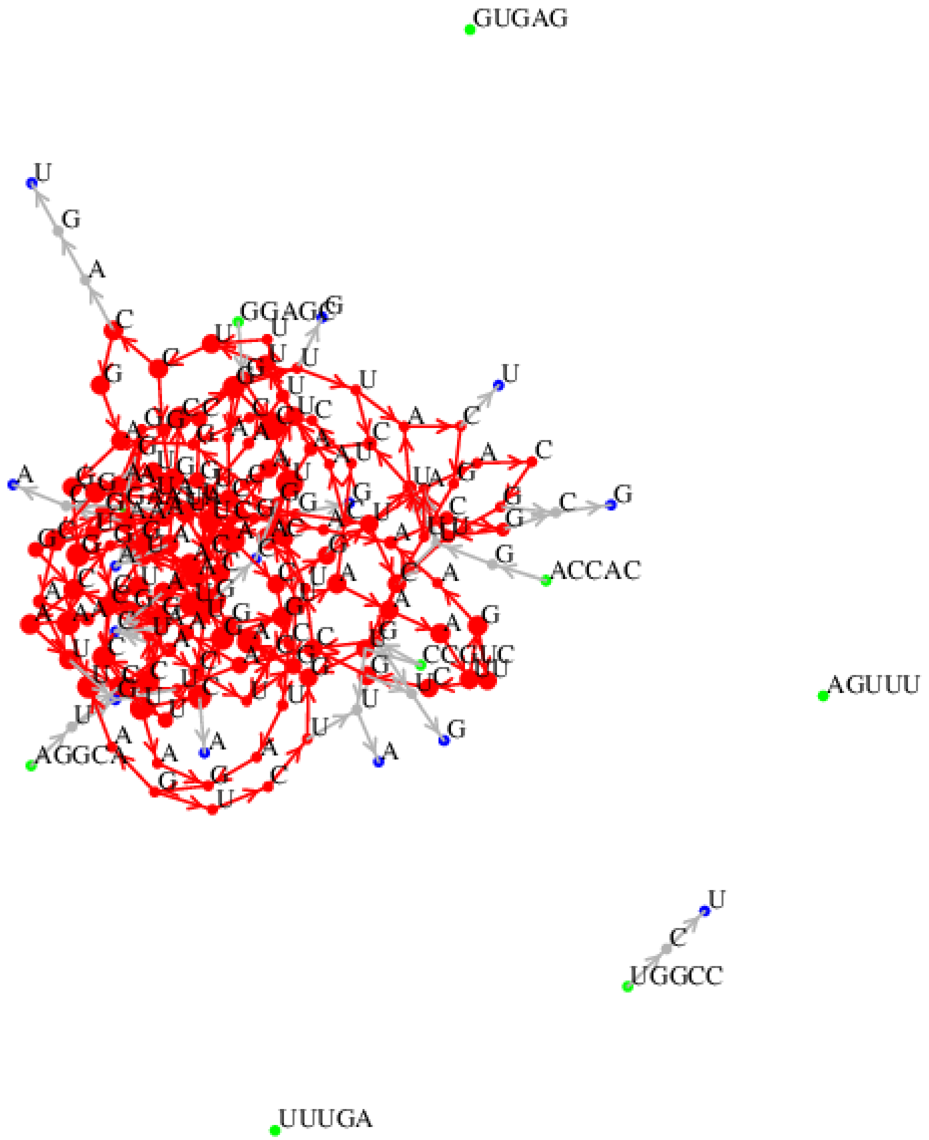

Fig S1.15: 40YC

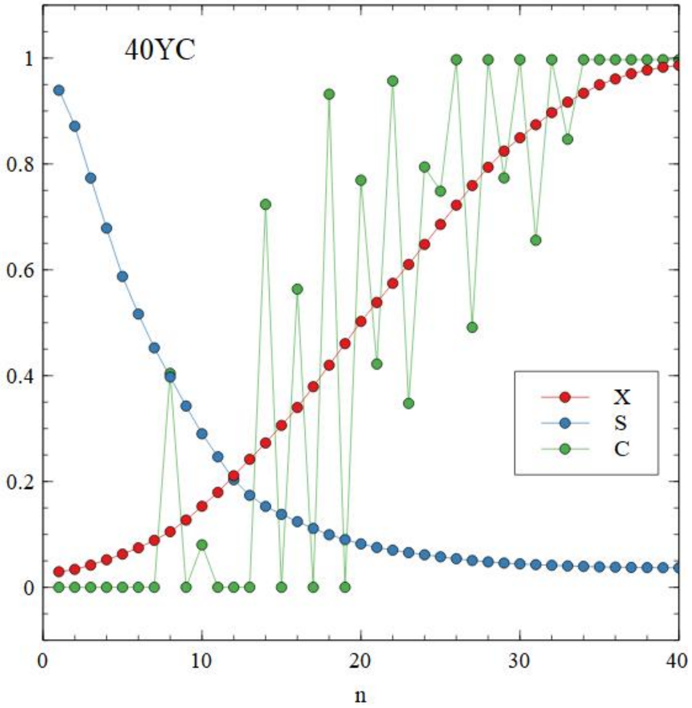

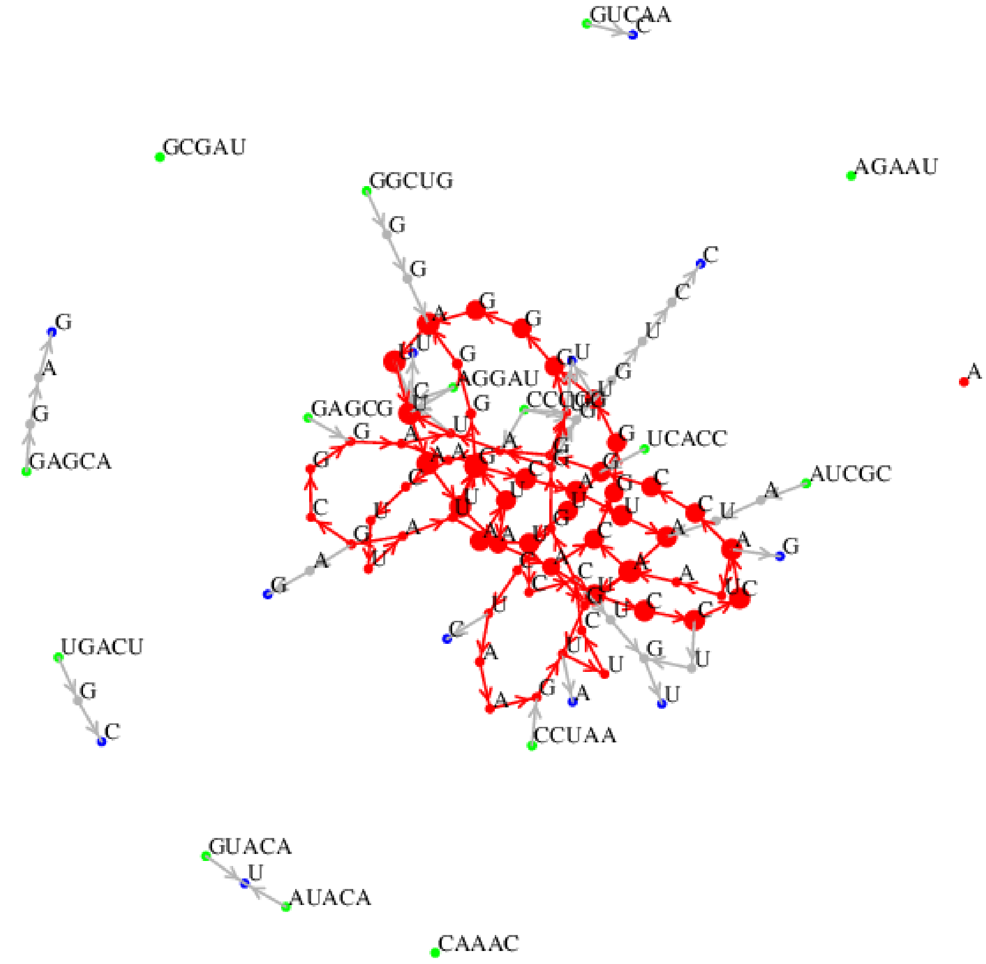

Fig S1.16: 40N1

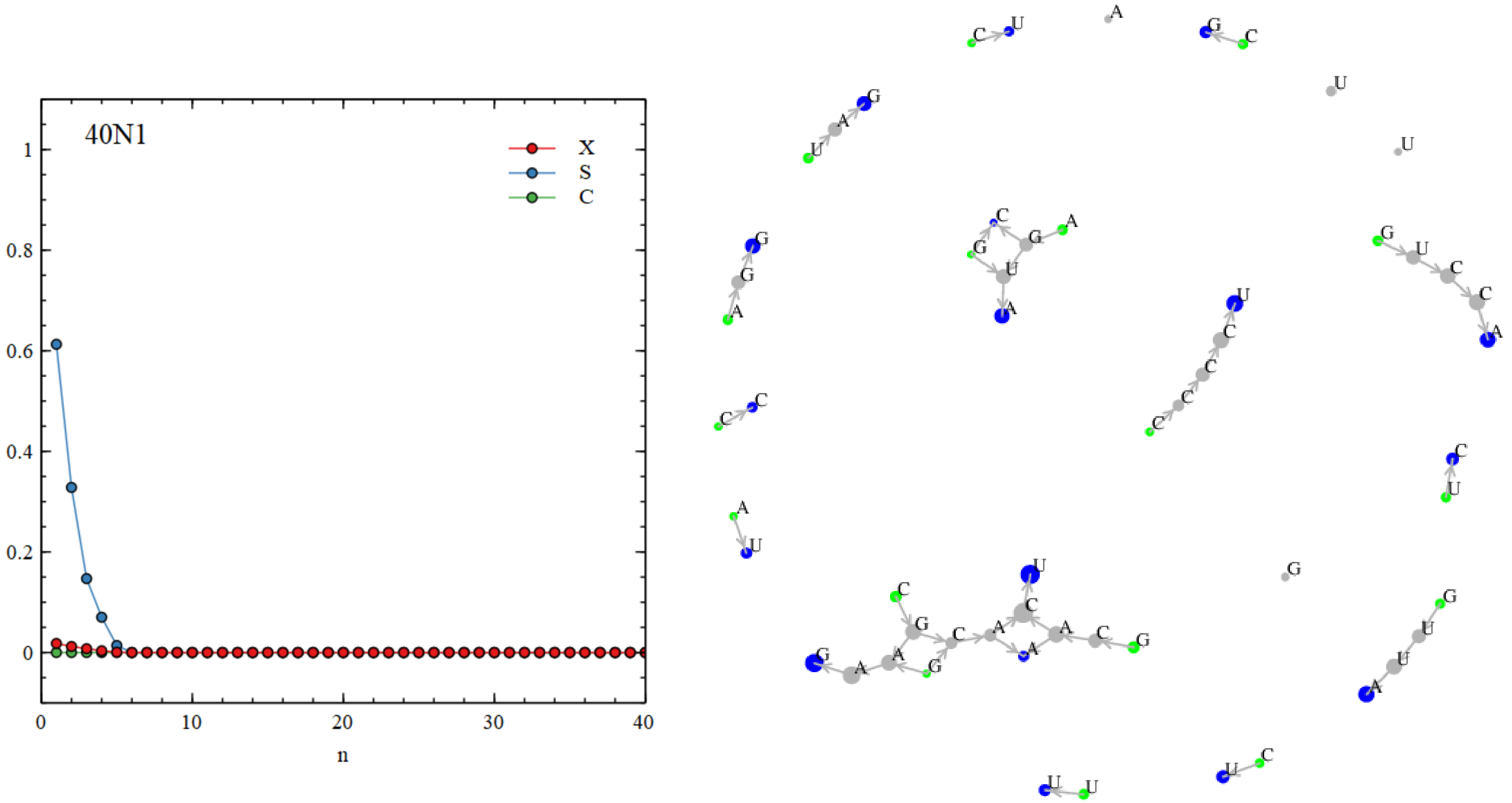

Fig S1.17: 40N2

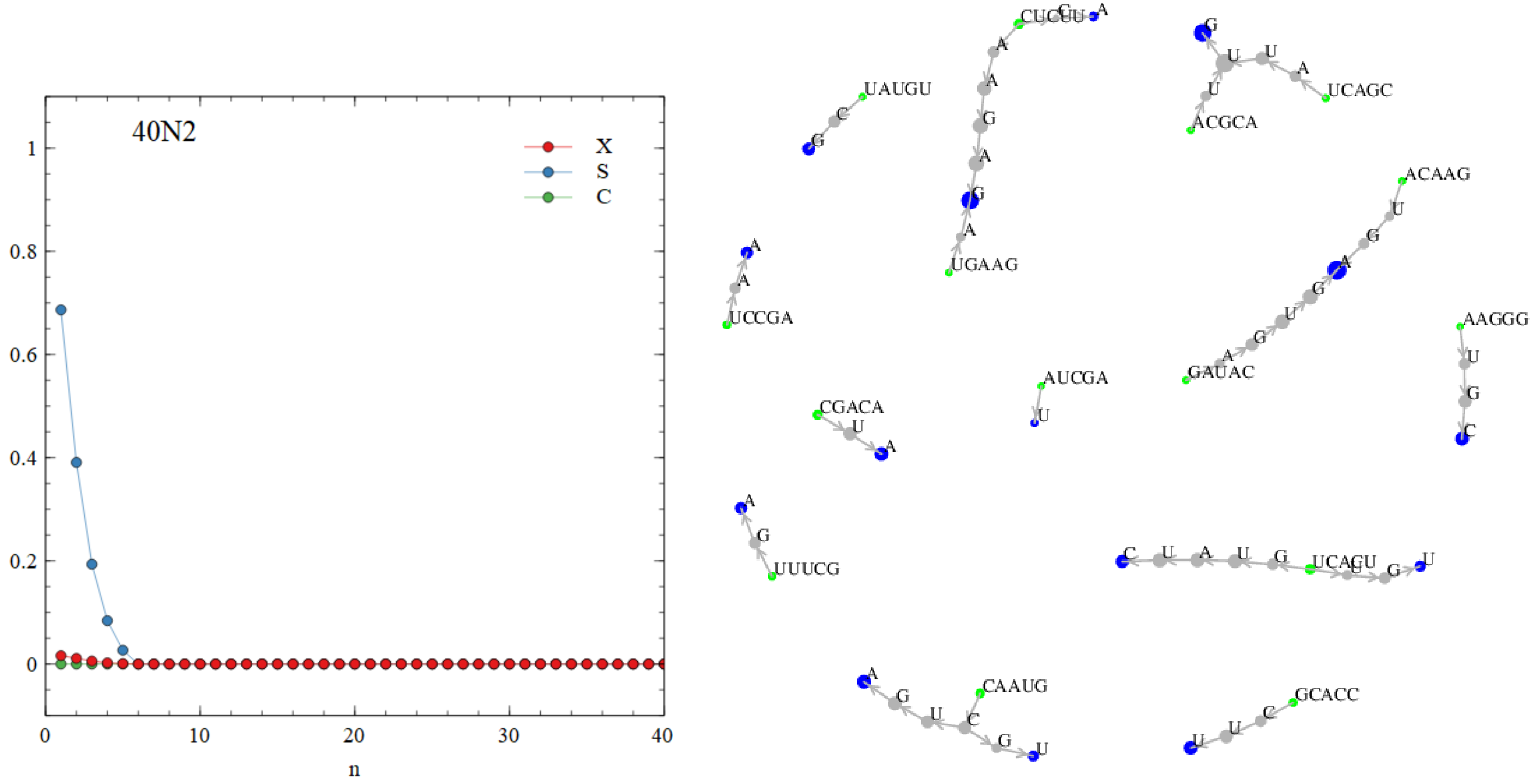

Fig S1.18: 40N3

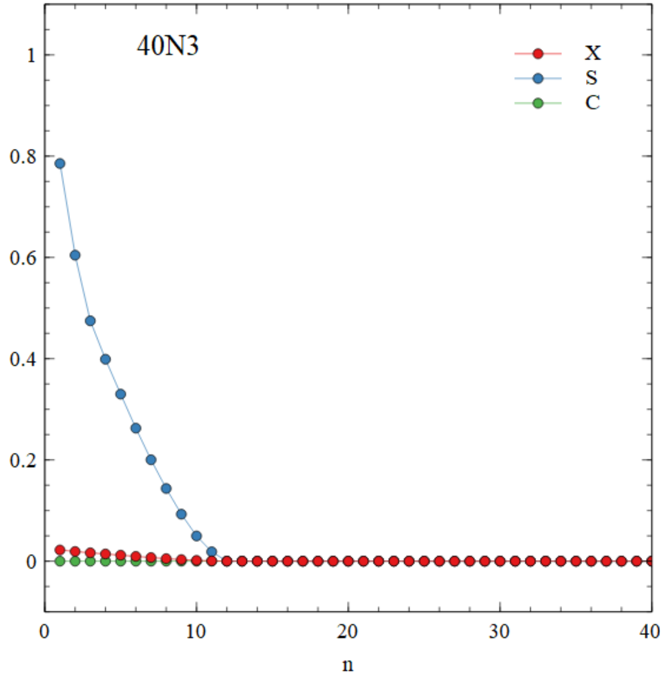

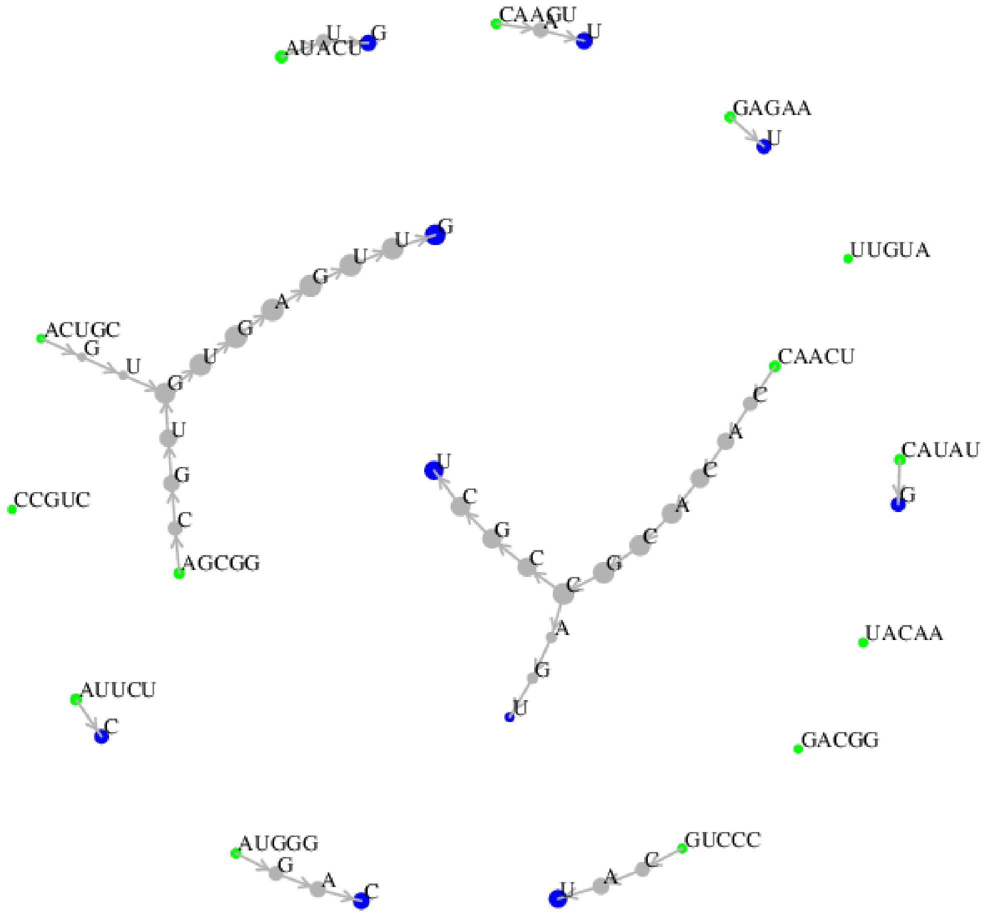

Fig S1.19: 40N4

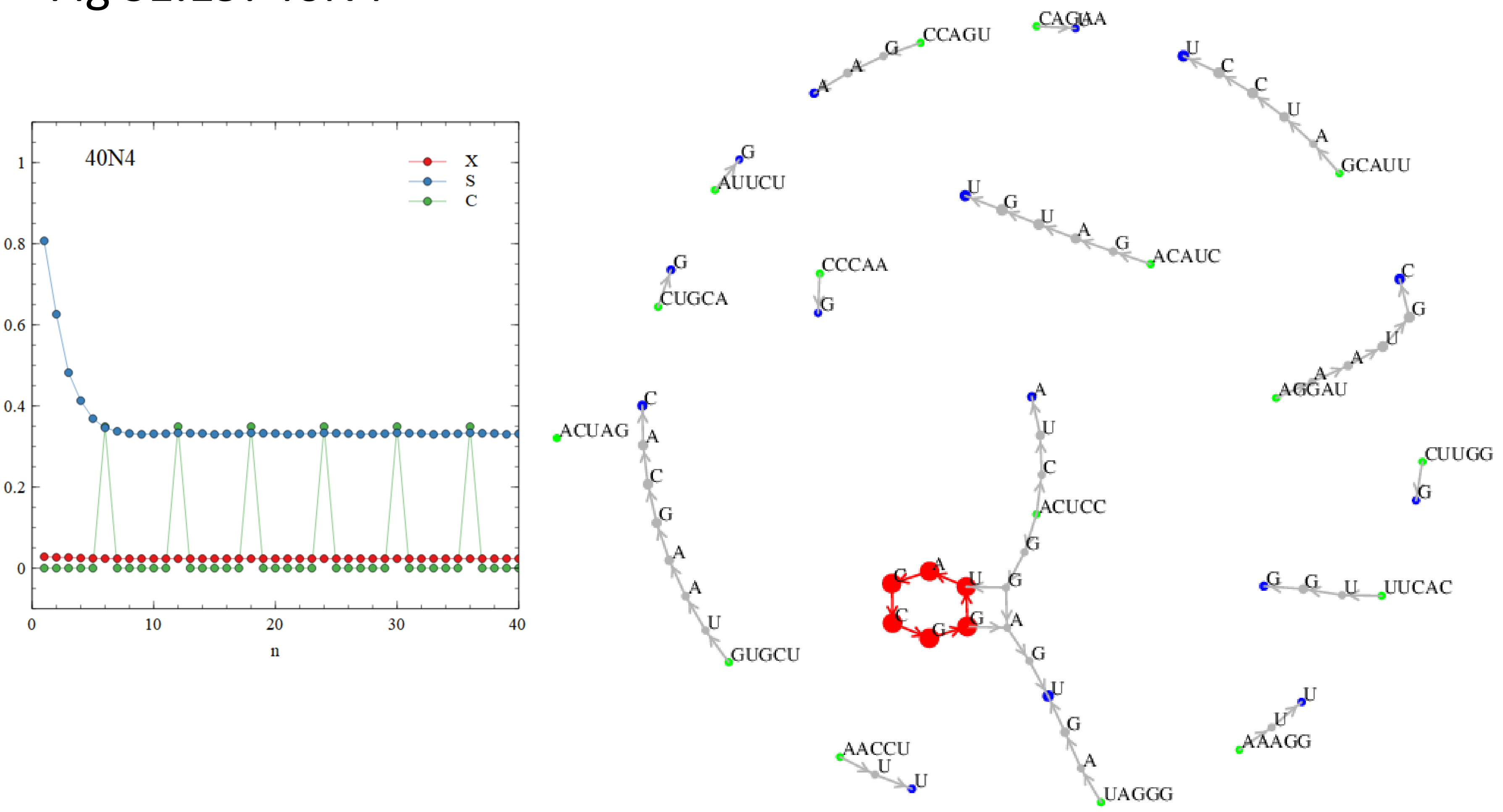

Fig S1.20: 40N5

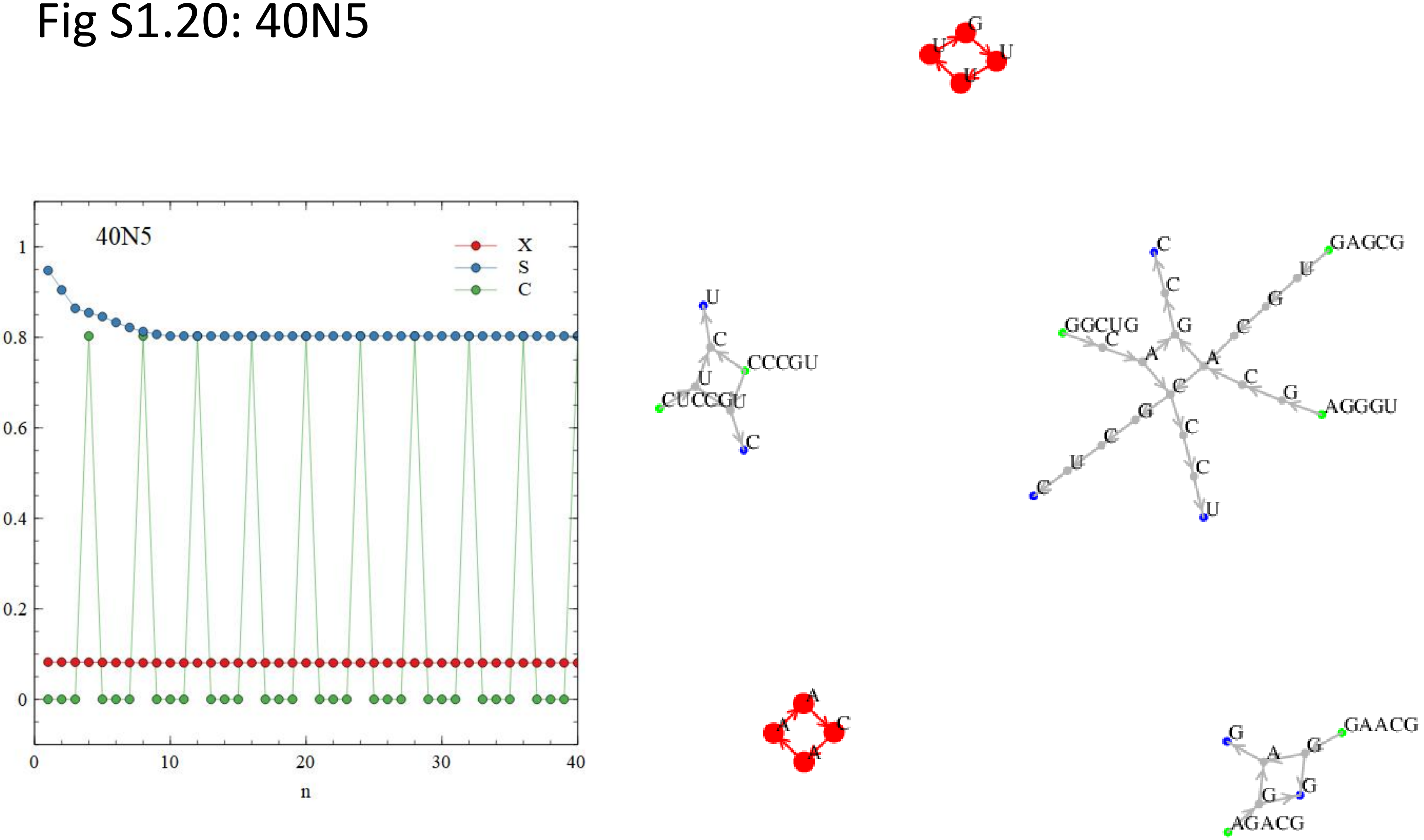

Fig S1.21: 40N6

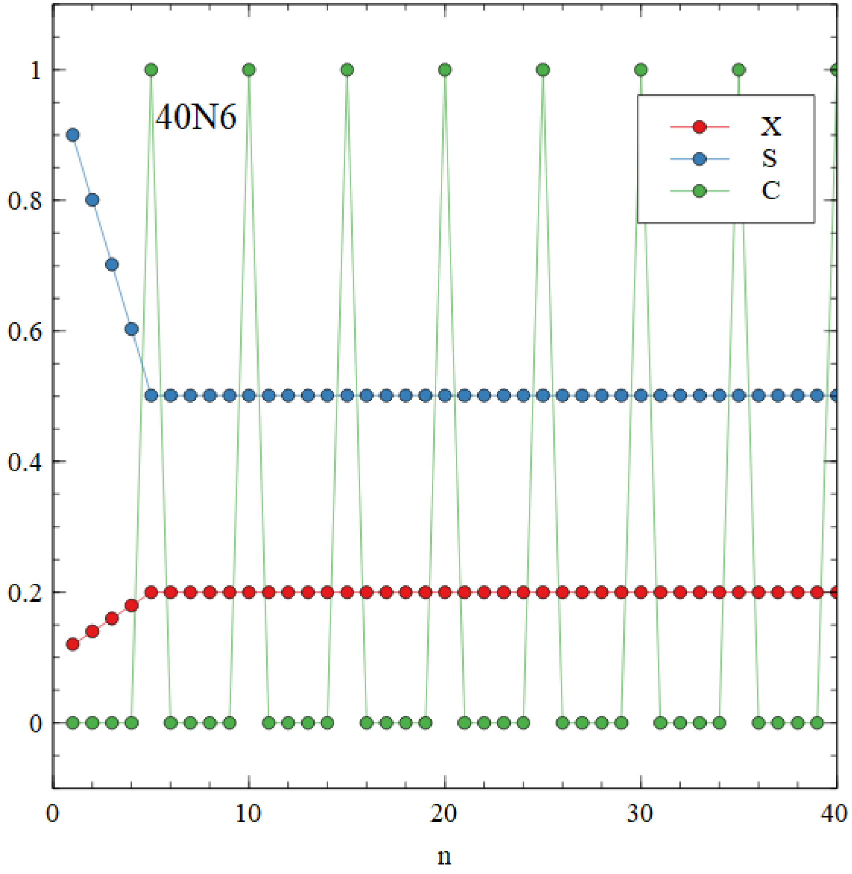

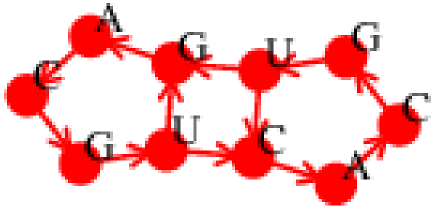

Fig S1.22: 40NC

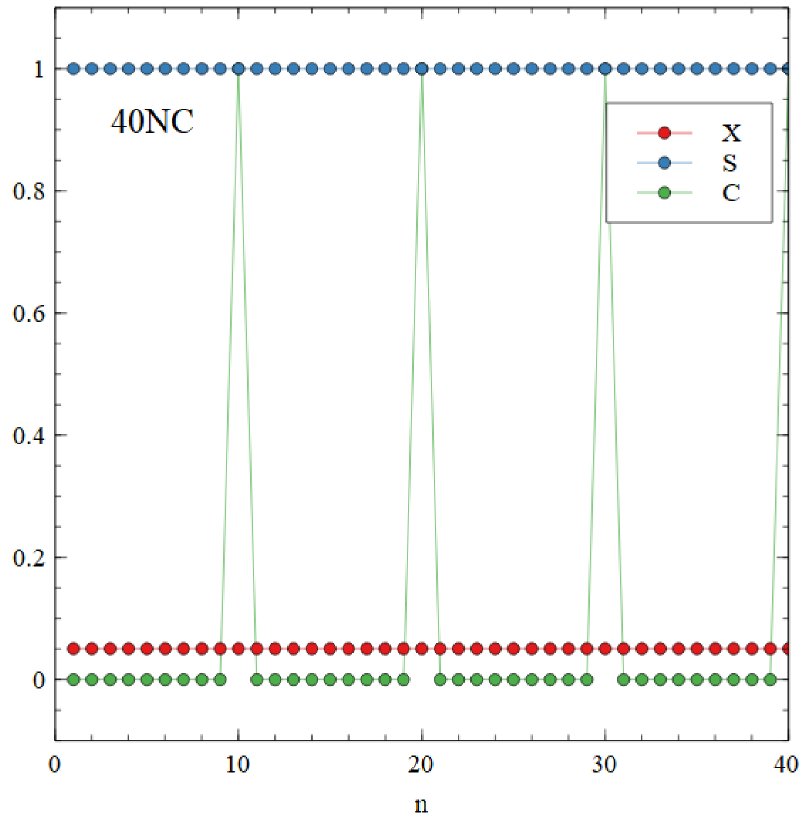

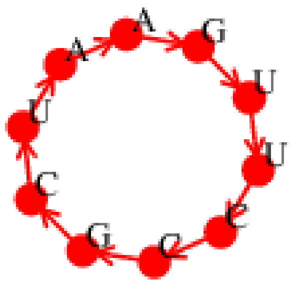

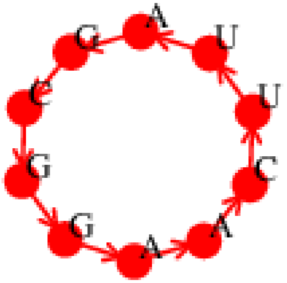

Supplement: S1 Fig — Each figure corresponds to one of the simulation runs in Table 5. For each run, the plot of the graph functions X(n), S(n) and C(n) is shown, together with the word graph. Parameters for each of the runs are described in the text in the section "Can a Virtual Circular Genome exist in our simulations?". (PDF) [file pcbi.1010458.s001.pdf]
